# Machine Learning-Based Identification of Preoperative Psychological Distress and Its Association With Adverse Surgery-Related Outcomes: Evidence From the China Surgery and Anesthesia Cohort (CSAC)

**DOI:** 10.1155/da/3990416

**Published:** 2025-10-24

**Authors:** Can Hou, Yao Yang, Wenwen Chen, Lei Yang, Yu Zeng, Qian Li, Huan Song

**Affiliations:** ^1^Department of Anesthesiology and West China Biomedical Big Data Center, West China Hospital, Sichuan University, Chengdu, China; ^2^Med-X Center for Informatics, Sichuan University, Chengdu, China; ^3^West China Biomedical Big Data Center, West China Hospital, Sichuan University, Chengdu, China; ^4^Department of Anesthesiology, West China Hospital, Sichuan University, Chengdu, China; ^5^Laboratory of Anesthesia and Critical Care Medicine, National-Local Joint Engineering Research, West China Hospital, Sichuan University, Chengdu, China; ^6^Center of Public Health Sciences, Faculty of Medicine, University of Iceland, Reykjavík, Iceland

**Keywords:** adverse surgery-related outcomes, preoperative psychological distress, psychological distress patterns, unsupervised machine learning

## Abstract

**Background:**

Many patients experience psychological distress in the preoperative phase, whilst screening based on cut-off points of assessment scales showed limited value in predicting clinical postoperative adverse outcomes.

**Methods:**

To identify preoperative psychological distress and investigate their associations with adverse surgery-related outcomes, we included 16,662 patients from the China Surgery and Anesthesia Cohort (CSAC). We applied dimensionality reduction and unsupervised machine learning algorithms to classify participants into distinct psychological patterns. We then assessed the associations of machine learning-identified psychological patterns and traditional cut-off based psychological symptoms, with various adverse surgery-related outcomes, using logistic and linear regression models while adjusting for other relevant covariates.

**Results:**

We successfully established clustering algorithms for 16,298 participants, demonstrating strong consistency in pattern features. Six distinct psychological patterns among participants were identified, including one group with normal psychological functioning and five groups with varying levels of psychological distress. All identified psychological distress patterns were significantly associated with most surgery-related adverse outcomes, both in short-term (e.g., any within-hospital postoperative complication, odds ratios [ORs] = 1.24–1.30) and long-term (e.g., cognitive impairment at 12 months postsurgery, 1.29–2.35). In contrast, traditional cut-off-based methods identified only 266 patients with significant psychological symptoms, which showed no association with some key short-term outcomes (e.g., length of hospital stay and postoperative complication), though they remained linked to most long-term outcomes.

**Conclusions:**

Our findings demonstrate the effectiveness of machine learning in accurately identifying patients with preoperative psychological distress who may require clinical attention, highlighting the potential of these techniques to guide targeted preoperative interventions and ultimately improve surgical outcomes.

**Summary**

• Traditional fixed cut-off points on psychological scales may inadequately capture all patients experiencing preoperative psychological distress.
• The psychological distress patterns identified through machine learning were strongly associated with both short- and long-term adverse surgical outcomes, emphasizing the clinical relevance of these subgroups.
• Our study demonstrates that advanced machine learning based technique outperform traditional methods by effectively identifying a greater number of surgical patients with preoperative psychological distress and enabling subgroup classification for more personalized interventions.

## 1. Introduction

Both major disease diagnosis and elective surgery are notable stressful events, often resulting in a considerable proportion of patients experiencing significant psychological distress, particularly during the preoperative period [1–3]. For instance, a meta-analysis of 28 studies and 14,652 participants concluded that the global pooled estimate for preoperative anxiety was 48%, with variations by types of surgeries and geographical locations [4]. Prevalence rate for preoperative depression, though somehow lower, stood at a comparable level, ranging from 5% to 34% [5–8]. However, in contrast to the wealth of data available from Europe and North America, estimates derived from studies involving Chinese populations were scarce [4, 9], and often with small and unrepresentative study samples.

Further, although the presence of preoperative psychological symptoms, as identified using the tradition cut-off points of applied psychological scales, has been linked with several self-rated (i.e., subjective) adverse surgery-related outcomes, such as postoperative pain-related symptoms [10], postoperative psychological conditions [11], lower quality of life and satisfaction [12, 13], studies examining its associations with objective and critical surgery-related complications, such as postoperative infection and delayed wound healing, have yielded inconsistent results [14]. Given the established knowledge that psychological factors can influence numerous biological functions essential for postoperative recovery (e.g., wound healing [15] and the development of an excessive inflammatory response [16]), it raises the possibility that the existing cut-off based methods may not effectively identify patients with psychological symptoms requiring clinical attention. On the contrary, the application of nonselective psychological interventions before surgery has shown little impact on core surgical outcomes (e.g., length of hospital stays, complications, and mortality) [17, 18], underscoring the importance of implementing effective psychological screening to enhance the postoperative prehabilitation strategy.

Prior to surgery, patients' psychological responses are often transient and shaped by short-term stressors such as an upcoming procedure or a recent diagnosis, presenting as highly heterogeneous yet co-occurred psychological symptoms [19], which challenges the utility of screening tools designed for general populations. Furthermore, from a perioperative management perspective, identifying symptom-based subgroups that are actionable and amenable to targeted interventions may be more informative and clinically useful than relying on conventional cutoffs used for diagnostic purposes. Data-driven unsupervised machine learning techniques provide a possible solution for such a scenario, as it could discern the underlying structure within the data and automatically categorize individuals into an optimal number of groups that characterized by distinct profiles. Previous efforts have shown successful utilization of this method to uncover psychological abnormality groups with clinical relevance [20].

Leveraging data from the China Surgery and Anesthesia Cohort (CSAC), which recruited approximately 16,000 middle-aged Chinese surgery patients from four medical centers in China, the present study aimed to capture preoperative psychological distress patterns that with importance to short-, intermediate-, and long-term adverse surgery-related outcomes, through unsupervised machine learning approaches. Unlike prior studies using CSAC data that dichotomized psychological symptoms and examined their associations with individual outcomes [21, 22], we employ an unsupervised clustering pipeline on the complete item-level Patient Health Questionnaire 9 (PHQ-9) and Generalized Anxiety Disorder 7 (GAD-7) data. Such an endeavor may improve our understanding of the impact of preoperative psychopathology on surgery-related outcomes and holds promise for enhancing the development of more effective psychological screening and interventions for surgical patients.

## 2. Materials and Methods

### 2.1. Study Design

The CSAC is an ongoing multicenter and prospective cohort study that has been enrolling adults aged 40–65 years undergoing elective surgery and general anesthesia. The recruitment timeline and details about each collaborating center can be found in Supporting Information: Methods and cohort profile [23]. For the current study, we used data from four primary CSAC centers: West China Hospital, West China Tianfu Hospital, the Second Hospital of Hebei Medical University, and the First People's Hospital of Longquanyi District. The inclusion criteria across all research centers of the CSAC is: patients aged 40–65 years scheduled for elective surgery under general anesthesia. We excluded nonlocal residents, patients undergoing day surgeries (the length of hospital admission < 2 days) or craniotomy, patients with less than primary-school education, and those unable to understand the informed consent form or study instruments. Particularly, the CSAC is characterized by its well-designed data collection process, with data quality ensured through a 14-day training program for full-time data collectors and a comprehensive, multiple-step protocol for data quality control. Both baseline and active follow-up data were collected by trained data collectors through face-to-face interviews (for baseline assessment and follow-up assessment on postoperative days 1, 3, and 7) or telephone interviews (for follow-up assessments at 1, 3, 6, and 12 months after surgery). Additionally, through periodical data linkage, information regarding intraoperative status and perioperative medical care was obtained from the electronic medical record (EMR) system. As of June 2024, a total of 18,520 eligible surgery patients were invited to participate in the study, among which 18,455 were successfully recruited in the study (response rate = 99.65%).

In the present study, we excluded patients who underwent cardiac surgeries (*n* = 1430, Supporting Information 3: Figure S1), as this specific population has distinct basic and clinical characteristics, as well as a higher incidence of postoperative adversities, compared with other noncardiac surgery patients. Among the remaining 17,025 patients, we further excluded 283 patients who were receiving antipsychotic therapy in the preoperative period to avoid potential confounding from ongoing treatments. We also excluded patients with incomplete baseline psychological assessment data (*n* = 18) and those recruited from nonprimary centers, resulting in 16,553 patients included in the analyses of psychological pattern identification. The association analyses were performed among 16,186 participants, due to the exclusion of outliers that recognized as the noise (i.e., did not have to belong to any identified pattern) by Uniform Manifold Approximation and Projection (UMAP)-Hierarchical Density-Based Spatial Clustering of Applications with Noise (HDBSCAN) method ("Statistical analyses" shows the details).

#### 2.1.1. Measurement of Preoperative Psychological Status

Anxiety and depression are primary manifestations of preoperative psychological distress [4, 6]. Accordingly, we assessed preoperative psychological status 1 day before surgery using self-administered psychological scales Patient Health Questionnaire 9 (PHQ-9) [24] for depression and Generalized Anxiety Disorder 7 (GAD-7) [25] for anxiety, respectively. Each item on both the PHQ-9 and GAD-7 was scored on a scale ranging from 0 (not at all) to 3 (nearly every day).

Of 16,553 participants, 11,376 patients enrolled between July 2020 and June 2023 were used as the dataset for exploring preoperative psychological patterns. Utilizing item-based scores (16 scores in total), we identified distinct psychological patterns using unsupervised machine learning methods ("Statistical analyses" provides the details). The accuracy of these clustering algorithms was validated in an independent dataset of 5177 participants enrolled between July 2023 and June 2024.

Additionally, based on the widely used cut-off points [26, 27], we identified patients with preoperative depressive or anxiety symptoms if their PHQ-9 total score ≥ 10 or GAD-7 total score ≥ 10, respectively.

#### 2.1.2. Ascertainment of Adverse Surgery-Related Outcomes

The short-term adverse outcomes of interest in this study encompassed postoperative length of stay, anesthesia-related discomfort, and surgery-related complications. To evaluate anesthesia-related discomfort, we assessed the subjective intensity of pain during resting and movement, as well as nausea and vomiting symptoms after surgery. These assessments were conducted at 1 and 3 days postoperatively using an 11-point Numeric Rating Scale (NRS) [28]. Surgery-related complications (Supporting Information 3: Table S1 provides the full list) were ascertained by a trained research coordinator through a manual review of medical records in the EMRs system following standard procedures outlined in the European Perioperative Clinical Outcome (EPCO) guidelines [29].

Regarding intermediate- and long-term adverse outcomes, we focused on postoperative cognitive dysfunction, short-term memory deterioration, sleep disturbance, as well as self-reported postoperative recovery and life satisfaction, all of which were assessed at 1- (intermediate), 6-, and 12-months (long-term) postsurgery. Cognitive dysfunction was defined as a total score <2 on the 8-Item Informant Interview to Differentiate Aging and Dementia (AD8) [30], while short-term memory deterioration was defined by the inability to recall all three words in the Three-word Recall Test [31]. We measured sleep quality using Pittsburgh Sleep Quality Index (PSQI), with a total score >5 considered as sleep disturbance [32]. Finally, for the evaluation of postoperative recovery and life satisfaction, we utilized a self-rated 11-point and a 4-point NRS, respectively (Supporting Information 3: Table S1).

#### 2.1.3. Covariates

We collected data on sociodemographic factors (including age, sex, and education) and lifestyle factors (including smoking status and current alcohol consumption) using touchscreen questionnaires administered during the baseline assessment. Additionally, we calculated the body mass index (BMI) based on self-reported height and weight. Moreover, information regarding surgery and anesthesia was directly extracted from the EMRs. Considering the known associations between baseline somatic comorbidities and surgery-related outcomes, we further calculated the Charlson [33] comorbidity index (CCI) for each participant based on their medical history of somatic diseases reported during the initial baseline assessment.

### 2.2. Statistical Analyses

#### 2.2.1. Identification of Preoperative Psychological Patterns

Using the exploration dataset of 11,376 participants, we utilized a well-established unsupervised machine learning pipeline [34] to analyze preoperative item scores of PHQ-9 and GAD-7, with the aim of identifying preoperative psychological patterns. First, nonlinear dimensionality reduction was conducted using the UMAP algorithm [35]. UMAP is known for its ability to generate low-dimensional features while preserving both local and global structures within the data [35]. This process generates a meaningful embedding where the density of points reflects the modularity of input features. Subsequently, clustering was performed based on these reduced features using the HDBSCAN algorithm [36]. This density-based clustering method is advantageous for not require prespecifying the number of clusters, allowing the optimal number of patient patterns to emerge from the data structure itself; and it can identify and label individuals who do not fit well into any cluster as “noise,” preventing the forced classification of atypical cases. Our initial step involved clustering all the GAD-7 and PHQ-9 items for the identification of distinct symptoms clusters which resulted in four symptom clusters of varying size. To ensure that clusters defined by more items did not dominate the analysis, we then assigned different weights to each item to ensure equal importance for each identified symptom cluster. Following this reweighting, we applied the pipeline to the reweighted GAD-7 and PHQ-9 item scores to categorize the participants into distinct groups (labeled by its most featured item scores). To ensure sufficient statistical power for subsequent association analyses, we merged the group with a sample size smaller than 200 with their neighboring group, as determined by the dendrogram. The optimal hyperparameters for the UMAP and HDBSCAN algorithms were selected through grid search approaches, aimed at maximizing clustering performance as evaluated by the Silhouette Score and Dunn's Index, as well as visual inspection of the resulting embeddings. Specifically, for the initial symptom clustering stage, the UMAP parameters were: *n*_epochs = 500, *n*_components = 2, *n*_neighbors = 2, and min_dist = 0.01. Then the HDBSCAN parameter for min_Pts was set to 2. For the psychological distress pattern identification stage, the UMAP parameters were *n*_epochs = 500, *n*_neighbors = 30, *n*_components = 2, and min_dist = 0.01, and the HDBSCAN parameter for min_Pts was set to 95.

In addition to the UMAP-HDBSCAN methods, we applied traditional unsupervised machine learning techniques, including *K*-means [37] and hierarchical clustering [38], to identify preoperative psychological patterns. We then evaluated the performance of those models using the Silhouette Score [39] and Dunn's Index [40].

Furthermore, to assess the reliability of established clustering algorithms, we applied them to the validation dataset of 5177 patients. The accuracy of the identified psychological patterns was assessed by comparing the consistency of pattern characteristics between the exploration and validation dataset, using visualization approach [41].

#### 2.2.2. Association Analyses

We used logistic regression models (for binary outcomes) or linear regression models (for continuous outcomes) to investigate the associations of machine learning-identified psychological distress patterns and cut-off based psychological symptoms, with short-term as well as intermediate and long-term adverse surgery-related outcomes. These models were adjusted for several covariates, including age at baseline (as a continuous variable), sex (male or female), education (elementary and lower, junior school, senior/secondary school, college, and above), BMI (as a continuous variable), CCI (as a continuous variable), smoking status (current, never, or previous), current alcohol consumption (yes or no), surgery site (abdomen, head and neck, thorax, and others), and duration of surgery (as a continuous variable). For anesthesia-related discomfort outcomes, the models were further adjusted for the type of anesthesia. Notably, when examining the relationships between preoperative psychopathology and postoperative cognitive dysfunction and short-term memory deterioration, individuals who were already identified as having these conditions before surgery were excluded from the corresponding analysis. Furthermore, in addition to analyzing the overall population, we performed subgroup analyses by surgery site.

The current work has been reported in line with the Strengthening the Reporting of Cohort Studies in Surgery (STROCSS) criteria [42]. All the statistical analyses were conducted using R (version 4.2.2, R Foundation for Statistical Computing, Vienna, Austria) and Python 3.8 (Python Software Foundation Delaware, USA). A two-sided *p*-value of less than 0.05 was considered as the threshold of statistical significance.

## 3. Results

### 3.1. Demographic and Clinical Characteristics of the Study Population

We included a total of 16,553 patients recruited into the CSAC between July 2020 and June 2024. The average age at baseline for the patients was 52.27 years (standard deviation [SD] = 7.07), with the majority being females (58.3%, Table 1). Among these patients, 12,175 (73.6%) were recruited from West China Hospital, while the remaining were recruited from other medical centers. The characteristics of patients in the exploration dataset (*n* = 11,376) and the validation dataset (*n* = 5177) were generally comparable (Table 1). Similarly, patients recruited from West China Hospital and those from other centers showed few differences in characteristics (Supporting Information 3: Table S2).

In terms of surgery site, abdomen surgeries were the most common, accounting for 46.1% of the study population (*n* = 7631), followed by head and neck surgeries (*n* = 3413, 20.6%) and thorax surgeries (*n* = 2493, 15.1%). The major anesthesia type used was intravenous–inhalation combined anesthesia (*n* = 15,626, 94.4%). The overall incidence rate of any postoperative complications in the study population is 10.9% (*n* = 1797), with pulmonary complication (*n* = 1473, 8.9%) and postoperative infection (*n* = 962, 5.8%) being the most frequent types of postoperative complications.

### 3.2. Preoperative Psychological Patterns

**Figure 1:**
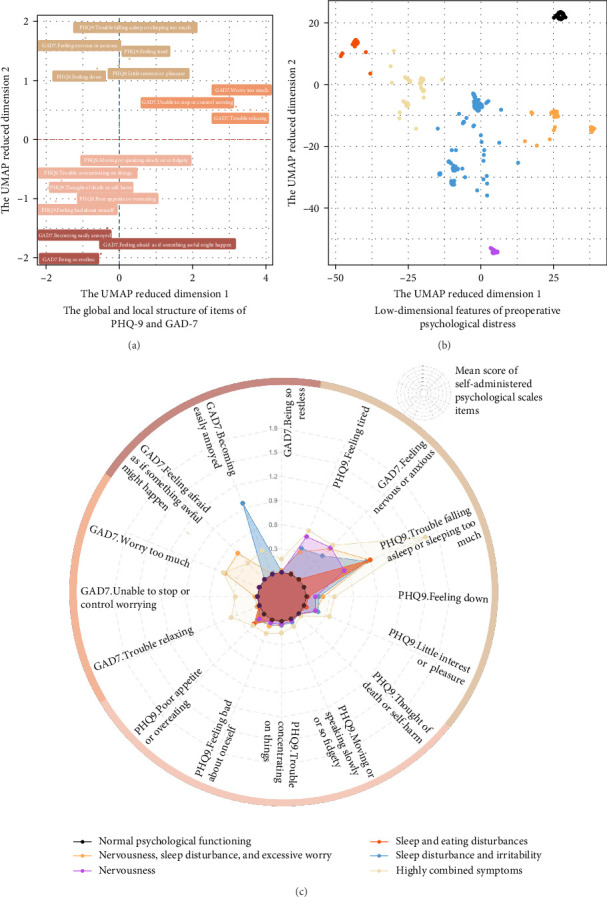
Preoperative psychological distress patterns identified using machine learning algorithms. UMAP, Uniform Manifold Approximation and Projection; HDBSCAN, Hierarchical Density-Based Spatial Clustering of Applications with Noise; GAD-7, Generalized Anxiety Disorder 7-Item Scale; PHQ-9, Patient Health Questionnaire 9. (A) Scatter plot showing the global and local structure of psychiatric scale items. Each dot represents an item of GAD-7 or PHQ-9. The *x*-axis and *y*-axis show the first and second UMAP-reduced features based on the item scores among all study populations. HDBSCAN further classified all the items into four distinct symptom clusters, each represented by a unique color in the plot. (B) Scatter plot showing the results of UMAP enhanced HDBSCAN for the identification of preoperative psychological distress patterns. Each dot represents a patient, with the *x*-axis and *y*-axis showing the first and second UMAP-reduced features based on the PHQ-9and GAD-7 item scores. HDBSCAN further classified all the patients into six distinct patterns (outliers were excluded), each represented by a unique color in the plot. (C) Radar chart showing the preoperative psychological characteristics (i.e., mean score of each PHQ-9 and GAD-7 item) across six distinct psychological groups identified using the UMAP enhanced HDBSCAN. Based on the extend and diversity of psychological symptoms, the six groups were labeled as “normal psychological functioning” (black), “highly combined symptoms” (gold), “nervousness” (purple), “sleep disturbance and irritability” (blue), “nervousness, sleep disturbance, and excessive worry” (orange), and “sleep and eating disturbances” (reddish-orange).

Applying the UMAP enhanced HDBSCAN method to the GAD-7 and PHQ-9 items in the exploration dataset yielded four symptom (i.e., item) clusters (Figure 1A), which led to the identification of six psychologically distinct groups within the study population, after dropping away outliers (2.2%) during the noise handling process (Figure 1B). These groups included one with normal psychological functioning (32.6%) and five exhibiting varying levels of psychological distress, named based on its extent and diversity of psychological symptoms as: “sleep and eating disturbance,” (13.2%) “sleep disturbance and irritability,” (3.2%) “nervousness,” (14.0%) “nervousness, sleep disturbance, and excessive worry,” (8.1%) and “highly combined symptoms,” (26.6%) respectively (Figure 1C). In comparison, the *K*-means and hierarchical clustering methods produced suboptimal results, as indicated by lower Silhouette Score and Dunn's Index values (Supporting Information 3: Table S3).

**Figure 2:**
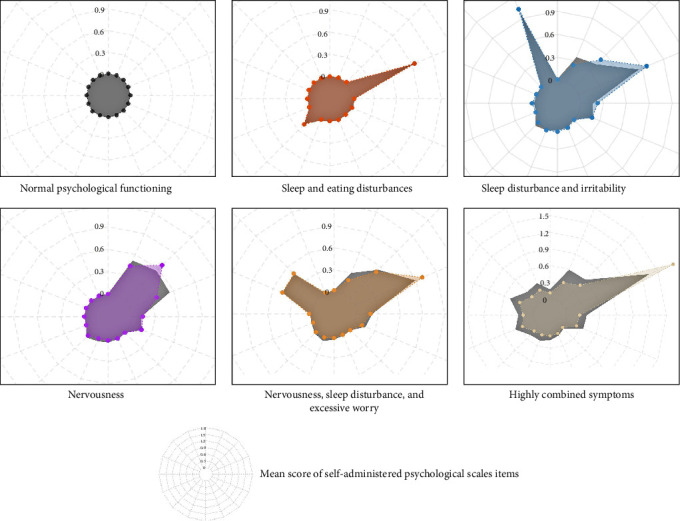
Comparison of preoperative psychological patterns in exploration and validation datasets. Radar chart showing the preoperative psychological characteristics (i.e., mean score of PHQ-9 and GAD-7 items) across six psychologically different groups for both exploration (in gray) and validation (in various other colors) datasets.

When the UMAP-HDBSCAN algorithms was applied to the validation dataset, they successfully identified six distinct preoperative psychological patterns, which strongly aligned with those identified in the exploration dataset in terms of symptom characteristics (Figure 2).

In the full analytic dataset for the association analyses (*n* = 16,186), 10,774 (66.6%) participants were classified into the five psychological distress groups. Supporting Information 3: Table S4 provides details on the number of participants in each group and their basic characteristics. Additionally, 266 patients (1.6%) were identified as experiencing depression or anxiety symptoms using traditional cut-off-based methods (Supporting Information 3: Table S4). Notably, females exhibited higher levels of psychological distress than males, as determined by either machine learning method (for the “nervousness, sleep disturbance, and excessive worry” pattern: 10.1% vs. 5.7%, Supporting Information 3: Table S5) or traditional cut-off-based classification (1.9% vs. 1.3%).

### 3.3. Associations Between Preoperative Psychopathology and Adverse Short-Term Surgery-Related Outcomes

**Figure 3:**
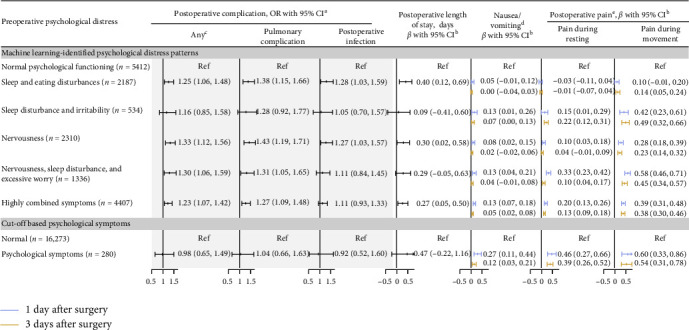
Associations between preoperative psychopathology and adverse short-term surgery-related outcomes. UMAP, Uniform Manifold Approximation and Projection; HDBSCAN, Hierarchical Density-Based Spatial Clustering of Applications with Noise; OR, odds ratio. ^a^ORs were derived from logistic regression models, adjusted for age, sex, body mass index, Charlson comorbidity index, smoking and drinking status, education level, surgical site, and duration of surgery. The reference group was the individuals with the “normal psychological functioning” pattern (for UMAP enhanced clustering method), or normal group (for traditional methods using cut-off value or hierarchy clustering). ^b^*β* (95% CIs) were derived from linear regression models, adjusted for age, sex, body mass index, Charlson comorbidity index, smoking and drinking status, education level, surgical site, and duration of surgery. All numeric values were standardized to a 0–10 scale. ^c^Short-term surgery-related complications were defined according to the European Perioperative Clinical Outcome (EPCO) Definitions. ^d^Nausea and vomiting symptoms after surgery were assessed at 1 day and 3 days postoperatively using an 11-point Numeric Rating Scale, with 0 indicating the absence of symptoms of nausea/vomiting and 10 representing the most severe symptoms of nausea/vomiting. ^e^Resting and movement pain after surgery were assessed at 1 day and 3 days postoperatively using an 11-point Numeric Rating Scale, with 0 indicating the absence of pain and 10 representing the most severe pain.

Concerning the duration of postoperative hospitalization, three, out of five, machine-learning identified psychological distress patterns exhibited statistically significant associations with an extended length of stay, compared to the normal psychological functioning group (i.e., the “sleep and eating disturbances,” “highly combined symptoms,” and “nervousness” patterns, *β*s = 0.27–0.40, Figure 3). Similarly, four psychological distress patterns stood out as they were significantly associated with an elevated risk of experiencing any postoperative complications, with highest estimates obtained for “nervousness, sleep disturbance, and excessive worry” (odds ratio [OR] = 1.30, 95% confidence interval [CI] 1.06–1.59) and “nervousness” (1.33, 1.12–1.56) patterns (Figure 3). Slightly lower but similar estimates were also noted for two major specific postoperative complications (i.e., pulmonary complication and infection, Figure 3). However, we did not observe any significant associations of cut-off based psychological symptoms with either prolonged postoperative hospitalization or any or specific postoperative complications (Figure 3).

Additionally, significant associations were observed between certain machine learning-identified psychological distress patterns and heightened postoperative discomforts (i.e., acute postoperative pain during rest or movement, and nausea and vomiting, Figure 3). Notably, the patterns of “nervousness, sleep disturbance, and excessive worry” and “highly combined symptoms” exhibited the most pronounced associations with pain during resting and movement, as well as nausea/vomiting at 1-day (*β*s = 0.13–0.58) postsurgery. Whereas the pattern of “sleep disturbance and irritability” showed strongest associations with pain during resting and movement, along with nausea/vomiting on day 3 (*β*s = 0.07–0.49) after surgery. Analyses on the cut-off based psychological status also found significant associations for those postoperative discomforts (*β*s = 0.27–0.60 for 1 day and 0.12–0.54 for 3 days after surgery, Figure 3).

### 3.4. Associations of Preoperative Psychopathology and Intermediate- and Long-Term Adverse Surgery-Related Outcomes

**Figure 4:**
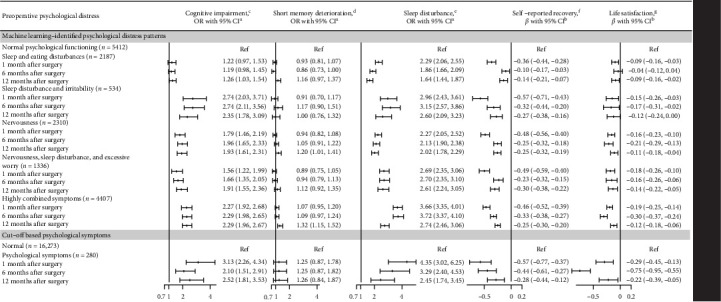
Associations between preoperative psychopathology and intermediate-and long-term surgery-related outcomes. UMAP, Uniform Manifold Approximation and Projection; HDBSCAN, Hierarchical Density-Based Spatial Clustering of Applications with Noise; OR, odds ratio. ^a^ORs were derived from logistic regression models, adjusted for age, sex, body mass index, Charlson comorbidity index, smoking and drinking status, education level, surgical site, and duration of surgery. The reference group was the individuals with the “normal psychological functioning” pattern (for UMAP enhanced clustering method), or normal group (for traditional methods using cut-off value or hierarchy clustering). ^b^*β* (95% CIs) were derived from linear regression models, adjusted for age, sex, body mass index, Charlson comorbidity index, smoking and drinking status, education level, surgical site, and duration of surgery. All numeric values were standardized to a 0–10 scale. ^c^Cognitive dysfunction was defined as a total score of less than 2 on the 8-Item Informant Interview to Differentiate Aging and Dementia (AD8). ^d^Short-term memory deterioration was defined by the inability to recall all three words in the Three-word Recall Test. ^e^Sleep disturbance as reporting a total of score of greater than 5 on the Pittsburgh Sleep Quality Index (PSQI). ^f^Self-reported postoperative recovery was employed using an 11-point NRS, with 0 indicating the worst recovery and 10 representing perfect recovery. ^g^Life satisfaction was assessed using a 4-point NRS, with 0 indicating total dissatisfaction and 4 representing total satisfaction.

A summary of the associations between preoperative psychopathology and intermediate- and long-term adverse surgery-related outcomes was illustrated in Figure 4. For cognitive-related outcomes, significant associations were observed between identified psychological distress patterns, particularly the “sleep disturbance and irritability” pattern (ORs = 2.35–2.74), and cognitive impairment across all assessment time points, whereas only “nervousness” and “highly combined symptoms” pattern was associated with short-term memory deterioration at 12 months postsurgery (ORs = 1.20–1.32). In addition, we observed significant associations of all preoperative psychological distress patterns with sleep disturbance, self-reported recovery and life satisfaction in intermediate- and long-term, with highest estimates consistently observed for the “highly combined symptoms” pattern (ORs = 2.74–3.72 for sleep disturbance, and *β*s = −0.46 to −0.25 and −0.30 to −0.12 for self-reported recovery and life satisfaction, respectively).

As for cut-off based psychological symptoms, the results were somehow similar (Figure 4)—except for short-term memory deterioration, significant associations were found for all studied intermediate- and long-term outcomes, with association magnitudes similar to those for the “highly combined symptoms” pattern (ORs = 2.10–3.13 for cognitive impairment, 2.45–4.35 for sleep disturbance, and *β*s = −0.57 to −0.28 for self-reported recovery and −0.75 to −0.22 for life satisfaction).

### 3.5. Subgroup Analyses

Although some variations in the magnitude of associations were observed, along with reduced statistical power in smaller subgroups, the overall risk patterns remained largely consistent across patients undergoing surgeries at different sites. (Supporting Information Figures S2 and S3).

## 4. Discussion

In the present study, through applying unsupervised machine learning techniques to the GAD-7 and PHQ-9 item scores collected preoperatively from more than 16,000 patients undergoing elective surgery, we successfully identified five groups of patients with distinct preoperative psychological distress patterns, and all of them demonstrated substantial adverse impacts on both short- and long-term surgery-related outcomes, particularly those measured objectively and considered important for clinical management (i.e., postoperative length of hospital stays and complications). Importantly, most of those patients with clinically relevant psychological distress did not meet the criteria for psychological symptoms when employing the traditional cut-off-based method (~1.6%). Besides further confirming the relationships between preoperative psychopathology and adverse surgery-related outcomes, our findings demonstrate the superiority of the machine learning-based approach over traditional methods in identifying patients with clinically significant preoperative psychological distress. If validated, this innovative tool promises to enhance clinical practices by enabling more precise identification of at-risk patients, thereby improving surgical outcomes. Moreover, the discovery of these heterogeneous patterns implies that a “one-size-fits-all” approach to perioperative psychological care is insufficient, emphasizing the need for personalized interventions tailored to specific psychological distress patterns.

Our current findings regarding the identification of five distinct preoperative psychological distress patterns and their relationships with adverse surgery-related outcomes are novel, with no comparable studies preceding this research. Nonetheless, these findings receive substantial support from numerous previous studies that has demonstrated links between preoperative psychological symptoms and postoperative adverse outcomes. Notably, prior research endeavors have employed different assessment scales and were predominantly based on the cut-off-based methods for identifying patients with psychological symptoms. Consequently, these studies have yielded some conflicting results, particularly with regard to objective outcomes [14], which typically hold greater clinical significance and warrant heightened attention. For instance, a longitudinal survey of 997 patients across 12 hospitals in China found that the presence of preoperative anxiety, measured by the State–Trait Anxiety Inventory, was significantly correlated with postoperative pain and poor sleep quality, but not with nausea or vomiting [43]. A European prospective cohort study indicated that preoperative distress assessed by the Beck Depression and Beck Anxiety Inventories was associated with increased postoperative complications, longer hospital stays, and higher rates of early readmission in females, but not in males [44]. Previous meta-analyses highlighted the large differences regarding conceptualization and measurement instruments of preoperative psychological conditions in various studies and found no clear association between preoperative anxiety or distress and most studied surgical outcomes [45, 46]. Indeed, in the present study, we also noted a lack of significant association between cut-off based psychological symptoms and postoperative complications, highlighting the importance of employing more advanced methods for identifying patients with preoperative psychopathology.

The “highly combined symptoms” pattern exhibited the most pronounced associations across most assessed outcomes. These results were expected, given that patients with this pattern typically experienced a wide range of preoperative psychological abnormities, which are likely to have the most significant adverse impacts [47]. Importantly, the patterns involved nervousness (i.e., “nervousness” and “nervousness, sleep disturbance, and excessive worry” patterns) also displayed strong associations with those outcomes, particularly the within-hospital ones (i.e., postoperative complication and length of hospital stay), to a level similar to the “highly combined symptoms” pattern. This may suggest a more profound and direct impact of specific anxiety-related symptoms (i.e., worrying and fear) compared with other symptoms on surgery-related outcomes, as supported by previous investigations [48]. Interestingly, for the remaining two patterns with shared sleep symptoms, we observed different results for different studied outcomes. Specifically, the pattern of “sleep and eating disturbances” was noted for its significant associations with within-hospital adverse outcomes, while the pattern of “sleep and irritability” showed notable associations with long-term outcomes such as cognitive impairment and reduced life satisfaction. Since within-hospital adverse outcomes are more closely associated with physical well-being, one speculation is that the manifestation of preoperative physical symptom (i.e., appetite issues) may introduce a more severe physical influence that matters for immediate medical outcomes, whereas manifestation of irritability symptoms may primarily signify an impact on mental well-being. Such speculation also gained some supports in some previous observational studies [49, 50], with however unclear biological mechanisms.

Our present study, which leverages high-quality longitudinal data from a multicenter hospital-based cohort of approximately 16,000 surgical patients, represents the largest study to date investigating the impact of preoperative psychopathology on postoperative adverse outcomes among Chinese patients. Other notable strengths of our study include the utilization of novel unsupervised machine learning techniques, an extended follow-up period of up to 12 months postsurgery, and the comprehensive assessment of a wide range of both objective and subjective adverse surgery-related outcomes. Importantly, while some identified clusters (e.g., the “nervousness” group) resemble clinically recognized constructs such as preoperative anxiety, many individuals in these groups had total GAD-7 scores below conventional thresholds. This suggests that a significant portion of at-risk patients may be missed by standard screening, underscoring the added clinical value of symptom-pattern–based approaches. Building on these findings, our team is now actively pursuing efforts to translate these data-driven insights into clinical practice, including the development and testing of pattern-specific interventions aimed at more effectively reducing surgery-related adverse outcomes than existing screening methods.

However, it is important to acknowledge certain limitations in our study that should be considered when interpreting the results. One significant limitation is the potential for uncontrolled confounding due to the heterogeneity of the included patients. The surgical patients in our study were recruited from various surgical specialties and underwent a diverse range of surgeries. While we have made efforts to address this issue by adjusting for variables such as the duration and type of surgery, residual confounding may still exist and could have contributed to the observed associations. Second, we relied exclusively on the PHQ-9 and GAD-7 to assess preoperative psychological status, which may not fully capture the entire spectrum of psychological conditions. Additionally, as these are self-reported measures, the use of physiological indicators such as heart rate variability [51] and salivary cortisol concentrations [52], to objectively assess psychological symptoms has gained increasing attention. Future studies may consider incorporating additional scales, such as the Self-Reporting Questionnaire (SRQ), and/or objective physiological markers to strengthen the robustness of our findings. Third, despite our efforts to construct two separate datasets for psychological pattern exploration and validation, the high homogeneity between those two populations (both from the CASC) might still raise concern about the accuracy and reliability of the clustering algorithms. This underscores the need of assessing the identified patterns in external populations. Fourth, our study primarily recruited middle-aged patients who underwent elective surgery, which consequently led to a lower incidence of some postoperative complications that are commonly observed among elderly patients (e.g., hallucination [53]). Associations between preoperative psychopathology and those rare outcomes need to be addressed in future studies. Last, it's worth noting that all the patients in our study were middle-aged individuals that admitted to medical centers located in the western region of China for elective surgery. As such, the generalizability of our results to other Chinese patients (e.g., patients with different age range, received emergence surgeries, or lived in other cities) needs cautions.

## 5. Conclusion

In conclusion, in the current study of approximately 16,000 middle-aged Chinese surgical patients, we established machine learning-based algorithms which can identify surgery patients with notable preoperative psychological distress that could considerably affect their both short-and long-term adverse surgery-related outcomes, particularly those with clinical importance. As the traditional cut-off-based approach showed inferior effectiveness, these findings underscore tailored psychological screening and intervention strategies for optimizing the preoperative management of surgery patients.

## Figures and Tables

**Table 1:** Characteristics of the patients included in the study.

Characteristics	Total, *N* = 16,553	Exploration dataset, *N* = 11,376	Validation dataset, *N* = 5177
Age at baseline (year), mean (SD)	52.27 (7.07)	52.33 (7.01)	52.14 (7.19)
Female, *n* (%)	9648 (58.3)	6681 (58.7)	2967 (57.3)
Body mass index (kg/m^2^), *n* (%)			
＜18.5	559 (3.4)	399 (3.5)	160 (3.1)
18.5–25	11,135 (67.3)	7723 (67.9)	3412 (65.9)
25–30	4365 (26.4)	2939 (25.8)	1426 (27.5)
≥30	494 (3.0)	315 (2.8)	179 (3.5)
Smoking status, *n* (%)			
Never	12,158 (73.4)	8340 (73.3)	3818 (73.7)
Previous smoker	1371 (8.3)	933 (8.2)	438 (8.5)
Current smoker	3014 (18.2)	2093 (18.4)	921 (17.8)
Unknown	10 (0.1)	10 (0.1)	0 (0.0)
Current alcohol consumption, *n* (%)			
No	13,550 (81.9)	9230 (81.1)	4320 (83.4)
Yes	3003 (18.1)	2146 (18.9)	857 (16.6)
Marital status, *n* (%)			
Unmarried	195 (1.2)	132 (1.2)	63 (1.2)
Married	15,708 (94.9)	10,743 (94.4)	4965 (95.9)
Divorced/widowed	650 (3.9)	501 (4.4)	149 (2.9)
Education level, *n* (%)			
Elementary and lower	502 (3.0)	351 (3.1)	151 (2.9)
Junior school	4173 (25.2)	2889 (25.4)	1284 (24.8)
Senior/secondary school	3630 (21.9)	2528 (22.2)	1102 (21.3)
Collage and above	8248 (49.8)	5608 (49.3)	2640 (51.0)
Occupation^a^, *n* (%)			
Manual laborers	1607 (9.7)	1072 (9.4)	535 (10.3)
Intellectual laborers	6471 (39.1)	4532 (39.8)	1939 (37.5)
Freelancer and self-employed	2266 (13.7)	1557 (13.7)	709 (13.7)
Retired	4879 (29.5)	3269 (28.7)	1610 (31.1)
Unemployed	1330 (8.0)	946 (8.3)	384 (7.4)
Type of anesthesia, *n* (%)			
Combined intravenous and inhalation anesthesia	15,626 (94.4)	10,755 (94.5)	4871 (94.1)
Total intravenous anesthesia	752 (4.5)	480 (4.2)	272 (5.3)
Inhalation anesthesia	109 (0.7)	109 (1.0)	0 (0.0)
Unknown	66 (0.4)	32 (0.3)	34 (0.7)
Anesthesia duration, *n* (%)			
≤90 min	3512 (21.2)	2297 (20.2)	1215 (23.5)
90–120 min	3203 (19.3)	2184 (19.2)	1019 (19.7)
121–180 min	4842 (29.1)	3376 (29.7)	1466 (28.3)
>180 min	4904 (29.6)	3459 (30.4)	1445 (27.9)
Unknown	92 (0.6)	60 (0.5)	32 (0.6)
Surgery duration, *n* (%)			
≤90 min	8493 (51.3)	5774 (50.8)	2719 (52.5)
90–120 min	2788 (16.8)	1920 (16.9)	868 (16.8)
121–180 min	3002 (18.1)	2077 (18.3)	925 (17.9)
>180 min	2194 (13.3)	1561 (13.7)	633 (12.2)
Unknown	76 (0.5)	44 (0.4)	32 (0.6)
Surgery site, *n* (%)			
Abdomen	7631 (46.1)	5297 (46.6)	2334 (45.1)
Head and neck	3413 (20.6)	2266 (19.9)	1147 (22.2)
Thorax	2493 (15.1)	1833 (16.1)	660 (12.7)
Others	3016 (18.2)	1980 (17.4)	1036 (20.0)
Moderate to severe preoperative psychological symptoms, *n* (%)			
No	16,273 (98.3)	11,169 (98.2)	5104 (98.6)
Yes (depression or anxiety symptoms)^b^	280 (1.7)	207 (1.8)	73 (1.4)
Postoperative ICU stay, *n* (%)			
No	16,237 (98.1)	11,114 (97.7)	5123 (99.0)
Yes	282 (1.7)	241 (2.1)	41 (0.8)
Unknown	34 (0.2)	21 (0.2)	13 (0.3)
Postoperative length of stay (day), mean (SD)	4.11 (5.68)	4.14 (4.19)	4.05 (8.03)
Postoperative complication, any types, *n* (%)			
No	14,723 (88.9)	10,078 (88.6)	4645 (89.7)
Yes	1797 (10.9)	1278 (11.2)	519 (10.0)
Unknown	33 (0.2)	20 (0.2)	13 (0.3)
Postoperative pulmonary complication, *n* (%)			
No	15,047 (90.9)	10,302 (90.6)	4745 (91.7)
Yes	1473 (8.9)	1054 (9.3)	419 (8.1)
Unknown	33 (0.2)	20 (0.2)	13 (0.3)
Postoperative infection, *n* (%)			
No	15,558 (94.0)	10,653 (93.6)	4905 (94.7)
Yes	962 (5.8)	703 (6.2)	259 (5.0)
Unknown	33 (0.2)	20 (0.2)	13 (0.3)

^a^Occupation was classified into five categories according to self-reported questionnaire: “manual laborers” incudes all workers and farmers; “intellectual laborers” includes students, civil servants, technicists, office clerks, managers, and servicemen; “freelancer and self-employed” includes freelancers and the self-employed individuals; “retired” refers solely to those who were retired; “unemployed” refers to those who are currently without a job.

^b^Depression or anxiety symptoms were defined as Patient Health Questionnaire-9 (PHQ-9) total score ≥10 or Generalized Anxiety Disorder Scale-7 (GAD-7) total score ≥10.

## Data Availability

CSAC database is not accessible publicly because of privacy policy and data protection regulation of patients. However, the authors welcome researchers to apply for the data access through contacting the corresponding author at songhuan@wchscu.cn, with proposed study objectives and proposals.

## Nomenclature

AD8: 8-Item Informant Interview to Differentiate Aging and Dementia
BMI: Body mass index
CCI: Charlson Comorbidity Index
CSAC: China Surgery and Anesthesia Cohort
EMRs: Electronic medical records
EPCO: European Perioperative Clinical Outcome
GAD-7: Generalized Anxiety Disorder 7
HDBSCAN: Hierarchical Density-Based Spatial Clustering of Applications with Noise
NRS: Numeric Rating Scale
OR: Odds ratio
PHQ-9: Patient Health Questionnaire 9
PSQI: Pittsburgh Sleep Quality Index
SD: Standard deviation
SRQ: Self-Reporting Questionnaire
STROCSS: Strengthening the Reporting of Cohort Studies in Surgery
UMAP: Uniform Manifold Approximation and Projection.

## Supporting Information

Supporting Information 1 The STROBE-checklist file provides the details about current study design, data collection, and statistical analysis, improving how our studies are reported, ultimately fostering better scientific communication and practical application.

Supporting Information 2 Supporting Information: Methods: We have provided more details about the CSAC in manuscript as well as in the Supporting Information: Methods, including research personnel involved, data-quality assurance, qualifications and staff turnover, inclusion and exclusion criterias, as well as detailed procedures for postoperative evaluations.

Supporting Information 3 Supporting Information: Figure S1 presents the detailed study flow chart illustrating the patient selection and data analysis process in the current study. Figures S2 and S3 depict the associations between preoperative psychopathology and adverse surgery-related outcomes (binary in S2 and continuous in S3) across different surgery sites, with color intensity indicating the strength of association. Table S1 provides a checklist of short-, intermediate- and long-term outcomes used in the current study. Table S2 compares the characteristics of the patients recruited from West China Hospital and those from other centers. Table S3 presents the Silhouette Score and Dunn's Index values for *K*-means, hierarchical clustering methods, and current UMAP-enhanced HDBSCAN methods. Table S4 compares the characteristics of the patients identified using current machine learning methods and traditional cut-off-based methods. Table S5 compares the characteristics of the patients between different sexes.

## References

[1] Hoang H., Laursen B., Stenager E. N., Stenager E. (2016). Psychiatric Co-Morbidity in Multiple Sclerosis: The Risk of Depression and Anxiety Before and After MS Diagnosis. *Multiple Sclerosis Journal*.

[2] Gallagher R., McKinley S. (2009). Anxiety, Depression and Perceived Control in Patients Having Coronary Artery Bypass Grafts. *Journal of Advanced Nursing*.

[3] Aust H., Eberhart L., Sturm T. (2018). A Cross-Sectional Study on Preoperative Anxiety in Adults. *Journal of Psychosomatic Research*.

[4] Abate S. M., Chekol Y. A., Basu B. (2020). Global Prevalence and Determinants of Preoperative Anxiety Among Surgical Patients: A Systematic Review and Meta-Analysis. *International Journal of Surgery Open*.

[5] Lunati M. P., Wilson J. M., Farley K. X., Gottschalk M. B., Wagner E. R. (2021). Preoperative Depression Is a Risk Factor for Complication and Increased Health Care Utilization Following Total Shoulder Arthroplasty. *Journal of Shoulder and Elbow Surgery*.

[6] Walker J., Burke K., Wanat M. (2018). The Prevalence of Depression in General Hospital Inpatients: A Systematic Review and Meta-Analysis of Interview-Based Studies. *Psychological Medicine*.

[7] Xu L., Pan Q., Lin R. (2016). Prevalence Rate and Influencing Factors of Preoperative Anxiety and Depression in Gastric Cancer Patients in China: Preliminary Study. *Journal of International Medical Research*.

[8] Bedaso A., Mekonnen N., Duko B. (2022). Prevalence and Factors Associated With Preoperative Anxiety Among Patients Undergoing Surgery in Low-Income and Middle-Income Countries: A Systematic Review and Meta-Analysis. *BMJ Open*.

[9] Liu X.-Y., Ma Y.-K., Zhao J.-C. (2018). Risk Factors for Preoperative Anxiety and Depression in Patients Scheduled for Abdominal Aortic Aneurysm Repair. *Chinese Medical Journal*.

[10] Pan X., Wang J., Lin Z., Dai W., Shi Z. (2019). Depression and Anxiety Are Risk Factors for Postoperative Pain-Related Symptoms and Complications in Patients Undergoing Primary Total Knee Arthroplasty in the United States. *The Journal of Arthroplasty*.

[11] McKenzie L. H., Simpson J., Stewart M. (2010). A Systematic Review of Pre-Operative Predictors of Post-Operative Depression and Anxiety in Individuals Who Have Undergone Coronary Artery Bypass Graft Surgery. *Psychology, Health and Medicine*.

[12] Muthukrishnan A., Tayyib N. A., Alsolami F. J., Ramaiah P., Lathamangeswaric C. (2023). Anxiety and Quality of Life Outcomes After Coronary Artery Bypass Graft Surgery - A Prospective Cohort Study. *Current Problems in Cardiology*.

[13] Hobson J. A., Slade P., Wrench I. J., Power L. (2006). Preoperative Anxiety and Postoperative Satisfaction in Women Undergoing Elective Caesarean Section. *International Journal of Obstetric Anesthesia*.

[14] Mavros M. N., Athanasiou S., Gkegkes I. D., Polyzos K. A., Peppas G., Falagas M. E. (2011). Do Psychological Variables Affect Early Surgical Recovery?. *PLoS ONE*.

[15] Broadbent E., Koschwanez H. E. (2012). The Psychology of Wound Healing. *Current Opinion in Psychiatry*.

[16] Glaus J., von Känel R., Lasserre A. M. (2018). The Bidirectional Relationship Between Anxiety Disorders and Circulating Levels of Inflammatory Markers: Results From a Large Longitudinal Population-Based Study. *Depression and Anxiety*.

[17] Tsimopoulou I., Pasquali S., Howard R. (2015). Psychological Prehabilitation Before Cancer Surgery: A Systematic Review. *Annals of Surgical Oncology*.

[18] Grimmett C., Heneka N., Chambers S. (2022). Psychological Interventions Prior to Cancer Surgery: A Review of Reviews. *Current Anesthesiology Reports*.

[19] Sorel J. C., Veltman E. S., Honig A., Poolman R. W. (2019). The Influence of Preoperative Psychological Distress on Pain and Function After Total Knee Arthroplasty: A Systematic Review and Meta-Analysis. *The Bone and Joint Journal*.

[20] Amoretti S., Verdolini N., Mezquida G. (2021). Identifying Clinical Clusters With Distinct Trajectories in First-Episode Psychosis Through an Unsupervised Machine Learning Technique. *European Neuropsychopharmacology*.

[21] Chen D., Yang H., Yang L. (2024). Preoperative Psychological Symptoms and Chronic Postsurgical Pain: Analysis of the Prospective China Surgery and Anaesthesia Cohort Study. *British Journal of Anaesthesia*.

[22] Yang L., Chen W., Yang D. (2023). Postsurgery Subjective Cognitive and Short-Term Memory Impairment Among Middle-Aged Chinese Patients. *JAMA Network Open*.

[23] Yang L., Chen W., Chen D. (2024). Cohort Profile: The China Surgery and Anesthesia Cohort (csac). *European Journal of Epidemiology*.

[24] Kroenke K., Spitzer R. L., Williams J. B. (2001). The Phq-9: Validity of a Brief Depression Severity Measure. *Journal of General Internal Medicine*.

[25] Spitzer R. L., Kroenke K., Williams J. B. W., Löwe B. (2006). A Brief Measure for Assessing Generalized Anxiety Disorder: The Gad-7. *Archives of Internal Medicine*.

[26] Sun Y., Fu Z., Bo Q., Mao Z., Ma X., Wang C. (2020). The Reliability and Validity of Phq-9 in Patients With Major Depressive Disorder in Psychiatric Hospital. *BMC Psychiatry*.

[27] Xiaoyan H., Chunbo L., Jie Q., Haisong C., Wenyuan W. (2010). Reliability and Validity of a Generalized Anxiety Disorder Scale in General Hospital Outpatients. *Shanghai Archives of Psychiatry*.

[28] Hartrick C. T., Kovan J. P., Shapiro S. (2003). The Numeric Rating Scale for Clinical Pain Measurement: A Ratio Measure?. *Pain Practice*.

[29] Jammer I., Wickboldt N., Sander M. (2015). Standards for Definitions and use of Outcome Measures for Clinical Effectiveness Research in Perioperative Medicine: European Perioperative Clinical Outcome (EPCO) Definitions: A Statement From the ESA-ESICM Joint Taskforce on Perioperative Outcome Measures. *European Journal of Anaesthesiology*.

[30] Galvin J., Roe C., Powlishta K. (2005). The AD8: A Brief Informant Interview to Detect Dementia. *Neurology*.

[31] Borson S., Scanlan J., Brush M., Vitaliano P., Dokmak A. (2000). The Mini-Cog: A Cognitive ‘Vital Signs’ Measure for Dementia Screening in Multi-Lingual Elderly. *International Journal of Geriatric Psychiatry*.

[32] Mollayeva T., Thurairajah P., Burton K., Mollayeva S., Shapiro C. M., Colantonio A. (2016). The Pittsburgh Sleep Quality Index as a Screening Tool for Sleep Dysfunction in Clinical and Non-Clinical Samples: A Systematic Review and Meta-Analysis. *Sleep Medicine Reviews*.

[33] Charlson M. E., Pompei P., Ales K. L., MacKenzie C. R. (1987). A New Method of Classifying Prognostic Comorbidity in Longitudinal Studies: Development and Validation. *Journal of Chronic Diseases*.

[34] Allaoui M., Kherfi M. L., Cheriet A. Considerably Improving Clustering Algorithms Using Umap Dimensionality Reduction Technique: A Comparative Study.

[35] McInnes L., Healy J., Melville J. (2018). Umap: Uniform Manifold Approximation and Projection for Dimension Reduction.

[36] González-Alemán R., Platero-Rochart D., Rodríguez-Serradet A. (2022). Mdscan: Rmsd-Based Hdbscan Clustering of Long Molecular Dynamics. *Bioinformatics (Oxford, England)*.

[37] Ahmed M., Seraj R., Islam S. M. S. (2020). The k-Means Algorithm: A Comprehensive Survey and Performance Evaluation. *Electronics*.

[38] Murtagh F., Contreras P. (2012). Algorithms for Hierarchical Clustering: An Overview. *WIREs Data Mining and Knowledge Discovery*.

[39] Shahapure K. R., Nicholas C. Cluster Quality Analysis Using Silhouette Score.

[40] Bezdek J. C., Pal N. R. (1998). Some New Indexes of Cluster Validity. *IEEE Transactions on Systems, Man and Cybernetics, Part B (Cybernetics)*.

[41] Gleicher M. (2018). Considerations for Visualizing Comparison. *IEEE Transactions on Visualization and Computer Graphics*.

[42] Mathew G., Agha R., Albrecht J. (2021). STROCSS 2021: Strengthening the Reporting of Cohort, Cross-Sectional and Case-Control Studies in Surgery. *International Journal of Surgery*.

[43] Li X.-R., Zhang W.-H., Williams J. P. (2021). A Multicenter Survey of Perioperative Anxiety in China: Pre- and Postoperative Associations. *Journal of Psychosomatic Research*.

[44] Geoffrion R., Koenig N. A., Zheng M. (2021). Preoperative Depression and Anxiety Impact on Inpatient Surgery Outcomes: A Prospective Cohort Study. *Annals of Surgery Open*.

[45] Yang K.-L., Detroyer E., Van Grootven B. (2023). Association Between Preoperative Anxiety and Postoperative Delirium in Older Patients: A Systematic Review and Meta-Analysis. *BMC Geriatrics*.

[46] Copeland L. A., Zeber J. E., Pugh M. J., Mortensen E. M., Restrepo M. I., Lawrence V. A. (2008). Postoperative Complications in the Seriously Mentally Ill: A Systematic Review of the Literature. *Annals of Surgery*.

[47] Hunt C., Slade T., Andrews G. (2004). Generalized Anxiety Disorder and Major Depressive Disorder Comorbidity in the National Survey of Mental Health and Well-Being. *Depression and Anxiety*.

[48] Ji W., Sang C., Zhang X., Zhu K., Bo L. (2022). Personality, Preoperative Anxiety, and Postoperative Outcomes: A Review. *International Journal of Environmental Research and Public Health*.

[49] McKernan M., McMillan D. C., Anderson J. R., Angerson W. J., Stuart R. C. (2008). The Relationship Between Quality of Life (eortc Qlq-c30) and Survival in Patients With Gastro-Oesophageal Cancer. *British Journal of Cancer*.

[50] Marek R. J., Le J. T., Hapenciuc G. (2024). Incremental Contribution of the Minnesota Multiphasic Personality Inventory – 3 to Predicting 1-Year Postoperative Spinal Cord Surgery/Spinal Cord Stimulation Outcomes. *Journal of Clinical Psychology in Medical Settings*.

[51] Amalan S., Vaishali B., S.P.P, Joseph J., Sivaprakasam M. Pre-surgery Stress Monitoring Using Heart Rate Variability Measures.

[52] Ferland C. E., Saran N., Valois T. (2017). Preoperative Distress Factors Predicting Postoperative Pain in Adolescents Undergoing Surgery: A Preliminary Study. *Journal of Pediatric Health Care*.

[53] Bowman E. M. L., Sweeney A. M., McAuley D. F. (2024). Assessment and Report of Individual Symptoms in Studies of Delirium in Postoperative Populations: A Systematic Review. *Age and Ageing*.

