# Supplementary material for: Machine Learning-Based Identification of Preoperative Psychological Distress and Its Association With Adverse Surgery-Related Outcomes: Evidence From the China Surgery and Anesthesia Cohort (CSAC)
Source: Depress Anxiety. 2025 Oct 24;2025:3990416. doi: 10.1155/da/3990416 (PMC12578553; doi:10.1155/da/3990416)
Supplement: Supporting Information 1 — The STROBE-checklist file provides the details about current study design, data collection, and statistical analysis, improving how our studies are reported, ultimately fostering better scientific communication and practical application. [file 3990416.f1.docx]

STROBE Statement—checklist of items that should be included in reports of observational studies

|  | | Item No. | | Recommendation | Page  No. | | Relevant text from manuscript |
| --- | --- | --- | --- | --- | --- | --- | --- |
| **Title and abstract** | | 1 | | (*a*) Indicate the study’s design with a commonly used term in the title or the abstract | 1 | | Title |
|  |  |  |  | (*b*) Provide in the abstract an informative and balanced summary of what was done and what was found | 2 | | Abstract section |
| Introduction | | | | | | |  |
| Background/rationale | | 2 | | Explain the scientific background and rationale for the investigation being reported | 4 | | Manuscript, lines 15-23,29-33 |
| Objectives | | 3 | | State specific objectives, including any prespecified hypotheses | 4, 5 | | Manuscript, lines 34-42 |
| Methods | | | | | | |  |
| Study design | | 4 | | Present key elements of study design early in the paper | 5 | | Manuscript, lines 46-47 |
| Setting | | 5 | | Describe the setting, locations, and relevant dates, including periods of recruitment, exposure, follow-up, and data collection | 5 | | Manuscript, lines 47-64 |
| Participants | | 6 | | (*a*) *Cohort study*—Give the eligibility criteria, and the sources and methods of selection of participants. Describe methods of follow-up  *Case-control study*—Give the eligibility criteria, and the sources and methods of case ascertainment and control selection. Give the rationale for the choice of cases and controls  *Cross-sectional study*—Give the eligibility criteria, and the sources and methods of selection of participants | 5 | | Manuscript, lines 51-60 |
|  |  |  |  | (*b*) *Cohort study*—For matched studies, give matching criteria and number of exposed and unexposed  *Case-control study*—For matched studies, give matching criteria and the number of controls per case |  | |  |
| Variables | | 7 | | Clearly define all outcomes, exposures, predictors, potential confounders, and effect modifiers. Give diagnostic criteria, if applicable | 6-7 | | Manuscript, lines 76-118 |
| Data sources/ measurement | | 8* | | For each variable of interest, give sources of data and details of methods of assessment (measurement). Describe comparability of assessment methods if there is more than one group | 6-7 | | Manuscript, lines 76-118  Supplementary Table1 |
| Bias | | 9 | | Describe any efforts to address potential sources of bias | 5-8 | | Manuscript, lines 55-57,65-71 |
| Study size | | 10 | | Explain how the study size was arrived at | 6 | | Manuscript, lines 65-72 Supplementary Figure S1 |
| Quantitative variables | 11 | | Explain how quantitative variables were handled in the analyses. If applicable, describe which groupings were chosen and why | | 8-9 | Manuscript, lines 124-125；135-136；161-165 | |
| Statistical methods | 12 | | (*a*) Describe all statistical methods, including those used to control for confounding | | 8-9 | Manuscript, lines 122-174 | |
|  |  |  | (*b*) Describe any methods used to examine subgroups and interactions | | 9 | Manuscript, lines 158-170 | |
|  |  |  | (*c*) Explain how missing data were addressed | | 6 | Manuscript, lines 69-70 | |
|  |  |  | (*d*) *Cohort study*—If applicable, explain how loss to follow-up was addressed  *Case-control study*—If applicable, explain how matching of cases and controls was addressed  *Cross-sectional study*—If applicable, describe analytical methods taking account of sampling strategy | |  |  | |
|  |  |  | (*e*) Describe any sensitivity analyses | |  |  | |
| Results | | | | | | | |
| Participants | 13* | | (a) Report numbers of individuals at each stage of study—eg numbers potentially eligible, examined for eligibility, confirmed eligible, included in the study, completing follow-up, and analysed | | 10 | Manuscript, lines 178-184 | |
|  |  |  | (b) Give reasons for non-participation at each stage | |  |  | |
|  |  |  | (c) Consider use of a flow diagram | |  | Supplementary Figure S1 | |
| Descriptive data | 14* | | (a) Give characteristics of study participants (eg demographic, clinical, social) and information on exposures and potential confounders | | 10 | Manuscript, lines 178-190  Table 1 | |
|  |  |  | (b) Indicate number of participants with missing data for each variable of interest | |  | Table 1 | |
|  |  |  | (c) *Cohort study*—Summarise follow-up time (eg, average and total amount) | |  |  | |
| Outcome data | 15* | | *Cohort study*—Report numbers of outcome events or summary measures over time | | *9* | Manuscript, lines 153-165  Table1 | |
|  |  |  | *Case-control study—*Report numbers in each exposure category, or summary measures of exposure | |  |  | |
|  |  |  | *Cross-sectional study—*Report numbers of outcome events or summary measures | |  |  | |
| Main results | 16 | | (*a*) Give unadjusted estimates and, if applicable, confounder-adjusted estimates and their precision (eg, 95% confidence interval). Make clear which confounders were adjusted for and why they were included | | 10-12 | Manuscript, lines 193-260 | |
|  |  |  | (*b*) Report category boundaries when continuous variables were categorized | |  |  | |
|  |  |  | (*c*) If relevant, consider translating estimates of relative risk into absolute risk for a meaningful time period | |  |  | |

Continued on next page

| Other analyses | 17 | Report other analyses done—eg analyses of subgroups and interactions, and sensitivity analyses |  |  |
| --- | --- | --- | --- | --- |
| Discussion | | | | |
| Key results | 18 | Summarise key results with reference to study objectives | 12 | Manuscript, lines 263-270 |
| Limitations | 19 | Discuss limitations of the study, taking into account sources of potential bias or imprecision. Discuss both direction and magnitude of any potential bias | 15 | Manuscript, lines 331-353 |
| Interpretation | 20 | Give a cautious overall interpretation of results considering objectives, limitations, multiplicity of analyses, results from similar studies, and other relevant evidence | 13-14 | Manuscript, lines 270-316 |
| Generalisability | 21 | Discuss the generalisability (external validity) of the study results | 15 | Manuscript, lines 349-353 |
| Other information | |  | | |
| Funding | 22 | Give the source of funding and the role of the funders for the present study and, if applicable, for the original study on which the present article is based | 17 | Acknowledgments, Funding section |

*Give information separately for cases and controls in case-control studies and, if applicable, for exposed and unexposed groups in cohort and cross-sectional studies.

**Note:** An Explanation and Elaboration article discusses each checklist item and gives methodological background and published examples of transparent reporting. Information on the STROBE Initiative is available at www.strobe-statement.org.
