# Supplementary material for: Machine Learning-Based Identification of Preoperative Psychological Distress and Its Association With Adverse Surgery-Related Outcomes: Evidence From the China Surgery and Anesthesia Cohort (CSAC)
Source: Depress Anxiety. 2025 Oct 24;2025:3990416. doi: 10.1155/da/3990416 (PMC12578553; doi:10.1155/da/3990416)
Supplement: Supporting Information 2 — Supporting Information: Methods: We have provided more details about the CSAC in manuscript as well as in the Supporting Information: Methods, including research personnel involved, data-quality assurance, qualifications and staff turnover, inclusion and exclusion criterias, as well as detailed procedures for postoperative evaluations. [file 3990416.f2.docx]

**Supplementary Methods**
***General information and study design***

The China Surgery and Anesthesia Cohort (CSAC) is an ongoing, multi-center prospective cohort study that was initiated in 2020. The primary objective of the study is to establish a comprehensive, high-quality database and biobank. This resource provides detailed multidimensional information for both hypothesis-driven and hypothesis-free research, covering lifestyle factors, somatic conditions, neuropsychological status, genetic data, and general anesthesia-related details. The study was first launched at West China Hospital in July 2020. This followed a pilot phase conducted between January 1st and July 14th, 2020, which served to optimize and standardize all procedures for data and biosample management. Subsequently, the study expanded to include three additional medical centers: The First People’s Hospital of Longquanyi District (since August 2021), West China Tianfu Hospital (since May 2022), and The Second Hospital of Hebei Medical University (since September 2022).

The study protocol and all subsequent amendments were approved by the Ethics Committee on Biomedical Research, West China Hospital of Sichuan University. The initial protocol was approved on April 21, 2020, with the approval number 2020-469. As the cohort evolved, two major amendments were reviewed and approved by the same ethics committee under the original approval number. The first amendment, approved on July 6, 2021, expanded the study population to include patients undergoing cardiac surgery, who had been previously excluded. The second amendment was approved on June 12, 2022, and it officially sanctioned the expansion of the study from a single-center to a multi-center cohort. This formally included collaborating sites such as The First People’s Hospital of Longquanyi District, West China Tianfu Hospital, The People's hospital of Neijiang Dongxing District, Incaier Sichuan Friendship Hospital, and The Second Hospital of Hebei Medical University. Written informed consent was obtained from all participants at all sites before their enrollment.

The inclusion criteria were applied uniformly across all participating centers. These criteria were: (1) patients aged between 40 and 65 years; (2) patients scheduled for elective surgery ; and (3) patients who agreed to receive general anesthesia during their operation. Patients were excluded if they met any of the following criteria: (1) were not residents of the city where the recruitment medical center was located; (2) were scheduled for day surgery, defined as a hospital admission of less than 2 days, or craniotomy; (3) had an education level below primary school; or (4) were unable to understand the interview scales. AInitially, the study focused on patients undergoing noncardiac surgeries, such as abdominal, thoracic, or otorhinolaryngologic procedures. Following the protocol amendment in July 2021, patients undergoing cardiac surgeries were also included in the cohort.

***Interview and Clinical Data collection***

At baseline (one day before surgery), trained staff conducted face-to-face interviews to collect data on sociodemographic, lifestyle, physical function, and preoperative psychological and cognitive status. The interviews were administered using touchscreen questionnaires on a custom-built Cohort Data Collection and Management System (CD-CMS, Version 1.0; ©West China Hospital). Biological samples were collected on the day of surgery prior to anesthesia.To create a comprehensive dataset, the study linked this collected information with clinical data from electronic medical resources. The Anesthesia Information Management System (AIMS) provided granular details of the surgery and anesthesia, including intraoperative events (e.g., hypotension, transfusion), dynamic vital signs, and drug administration. Concurrently, the Electronic Medical Record (EMR) system from each center supplied data on diagnoses, laboratory tests, imaging results, and other in-hospital care.

Participants were followed for one-year post-surgery. In-hospital assessments occurred face-to-face on days 1, 3, and 7 to evaluate immediate surgical complications, pain, and cognitive status. After discharge, follow-ups were conducted at 1, 3, 6, and 12 months via telephone or online questionnaires to track the trajectories of pain and monitor psychological and cognitive recovery. To maximize retention, at least two contact numbers for patients or their relatives were obtained during initial recruitment. For postoperative evaluations, data were collected only from patients who were conscious and not intubated. If a patient was unconscious, deeply sedated, or intubated at the time of a scheduled follow-up, the assessment was omitted, and the data were recorded as missing. These missing values were censored in statistical analyses.

***Biosample Collection and Processing***

On the day of surgery, peripheral blood (20 ml) and hair samples were collected with patient consent.

- Blood Samples: 10 ml of blood was collected into EDTA tubes for plasma and cell separation and immediately stored at 4°C. Another 10 ml was collected into clot activator serum separation tubes (SSTs), kept at room temperature for 30-45 minutes, then stored at 4°C. All blood samples were centrifuged at 3000 rpm for 15 minutes at 4°C within 4 hours of collection. The resulting plasma, cells, and serum were aliquoted into cryogenic vials and stored at -80°C.
- Hair Samples: A 3 cm sample of hair was cut from the root at a location 5 cm below the patient’s occipital tuberosity. Samples were stored in specimen bags with an identified code

***Data Quality Control***

A multi-faceted approach was implemented to ensure high data quality. All full‑time data collectors completed a standardized 14‑day training program designed to ensure they could accurately instruct patients on how to understand and independently complete the electronic questionnaires. A total of  97 full-time data collectors are employed across our four centers, with the number per site varying according to patient volume (see Figure 1). Each center also maintained a research and quality-control team composed of administrative staff and physicians who monitored the recruitment and data-collection process and ensured data quality. The average caseload per full-time collector ranged from 52 to 171 (median 110) patients per quarter, reflecting an optimal workload.

Data quality was first guaranteed by logical checks and extreme-value inquiries implemented in our electronic data collection system. Additionally, with patient consent, we audio-recorded each data‑collection session and conducted regular audits by the quality‑control team at each site. For routinely recorded clinical variables, such as occurrence of post-operative complications and infections, we implemented a pilot phase to harmonize ascertainment criteria across centers. Clinical data, including postoperative complications, are assessed by physicians during routine care and then directly extracted from each center’s information system.

## **Table 1. The timeline of current study involving other medical centers**

| Study center | Total cases | The start of the recruitment | The end of the recruitment |
| --- | --- | --- | --- |
| West-China hospital | 13887 | 2020-07-15 | Ongoing |
| West-China Tianfu hospital | 3802 | 2022-05-23 | Ongoing |
| The Second Hospital of HeBei Medical University | 303 | 2022-04-14 | Ongoing |
| The First People's hospital of Longquanyi District | 383 | 2021-07-29 | 2023-03-16 |


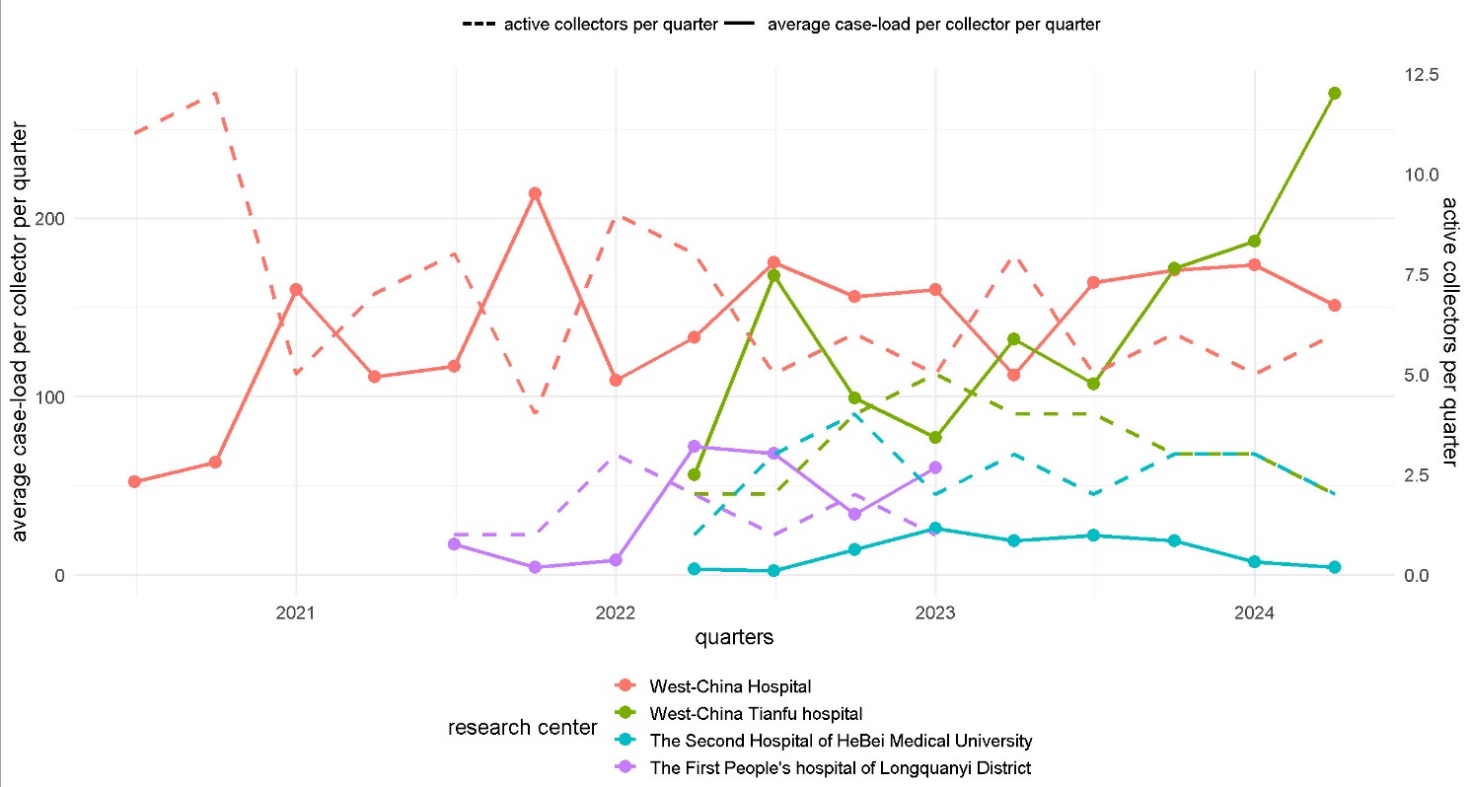


## **Figure 1 The dynamic number of data collectors and their average case load per quarters, by research centers**

The solid lines indicate the average case load of the data collectors per quarters, while the dash lines depict the number of active data collectors
