# Supplementary material for: Machine Learning-Based Identification of Preoperative Psychological Distress and Its Association With Adverse Surgery-Related Outcomes: Evidence From the China Surgery and Anesthesia Cohort (CSAC)
Source: Depress Anxiety. 2025 Oct 24;2025:3990416. doi: 10.1155/da/3990416 (PMC12578553; doi:10.1155/da/3990416)
Supplement: Supporting Information 3 — Supporting Information: Figure S1 presents the detailed study flow chart illustrating the patient selection and data analysis process in the current study. Figures S2 and S3 depict the associations between preoperative psychopathology and adverse surgery-related outcomes (binary in S2 and continuous in S3) across different surgery sites, with color intensity indicating the strength of association. Table S1 provides a checklist of short-, intermediate- and long-term outcomes used in the current study. Table S2 compares the characteristics of the patients recruited from West China Hospital and those from other centers. Table S3 presents the Silhouette Score and Dunn's Index values for K-means, hierarchical clustering methods, and current UMAP-enhanced HDBSCAN methods. Table S4 compares the characteristics of the patients identified using current machine learning methods and traditional cut-off-based methods. Table S5 compares the characteristics of the patients between different sexes. [file 3990416.f3.docx]

**Contents**

[Figure S1 Study flowchart 2](#_Toc207010238)

[Figure S2 Associations between preoperative psychopathology and adverse surgery-related binary outcomes, by surgery sites 3](#_Toc207010239)

[Figure S3 Associations between preoperative psychopathology and adverse surgery-related continuous outcomes, by surgery sites 4](#_Toc207010240)

[Supplementary table 1 Short-, intermediate- and long-term outcomes checklist 5](#_Toc207010241)

[Supplementary table 2. Characteristics of the patients, by study center 7](#_Toc207010242)

[Supplementary table 3 Evaluation of applied clustering methods 9](#_Toc207010243)

[Supplementary table 4 Characteristics of the patients, by preoperative psychiatric distress patterns 9](#_Toc207010244)

[Supplementary table 5 Sex specific characteristics of preoperative psychiatric distress patterns 13](#_Toc207010245)

**18,520 eligible surgery patients**

Excluded:

65 patients unwilling to participate

**18,455 patients** recruited in the CSAC

Excluded:

1,430 patients underwent cardiac surgeries

283 patients receiving antipsychotic therapy

171 patients collected from secondary centers (i.e., with number of patients less than 100)

18 patients with incomplete baseline psychological assessment data

**16,553 patients**

**Preoperative psychological patterns identification**

**Validation dataset:**

5,177 patients

**Exploration dataset:**

11,376 patients

enrolled between July 2020 and June 2023

enrolled between July 2023 and June 2024

Excluded:

367 patients that recognized as the outliers (i.e., did not have to belong to any identified pattern)

**Associations between preoperative psychopathology and adverse surgery-related outcomes**

**16,186 patients**

## Figure S1 Study flowchart

CSAC, The China Surgery and Anesthesia Cohort; UMAP, Uniform Manifold Approximation and Projection; HDBSCAN, Hierarchical Density-Based Spatial Clustering of Applications with Noise

**
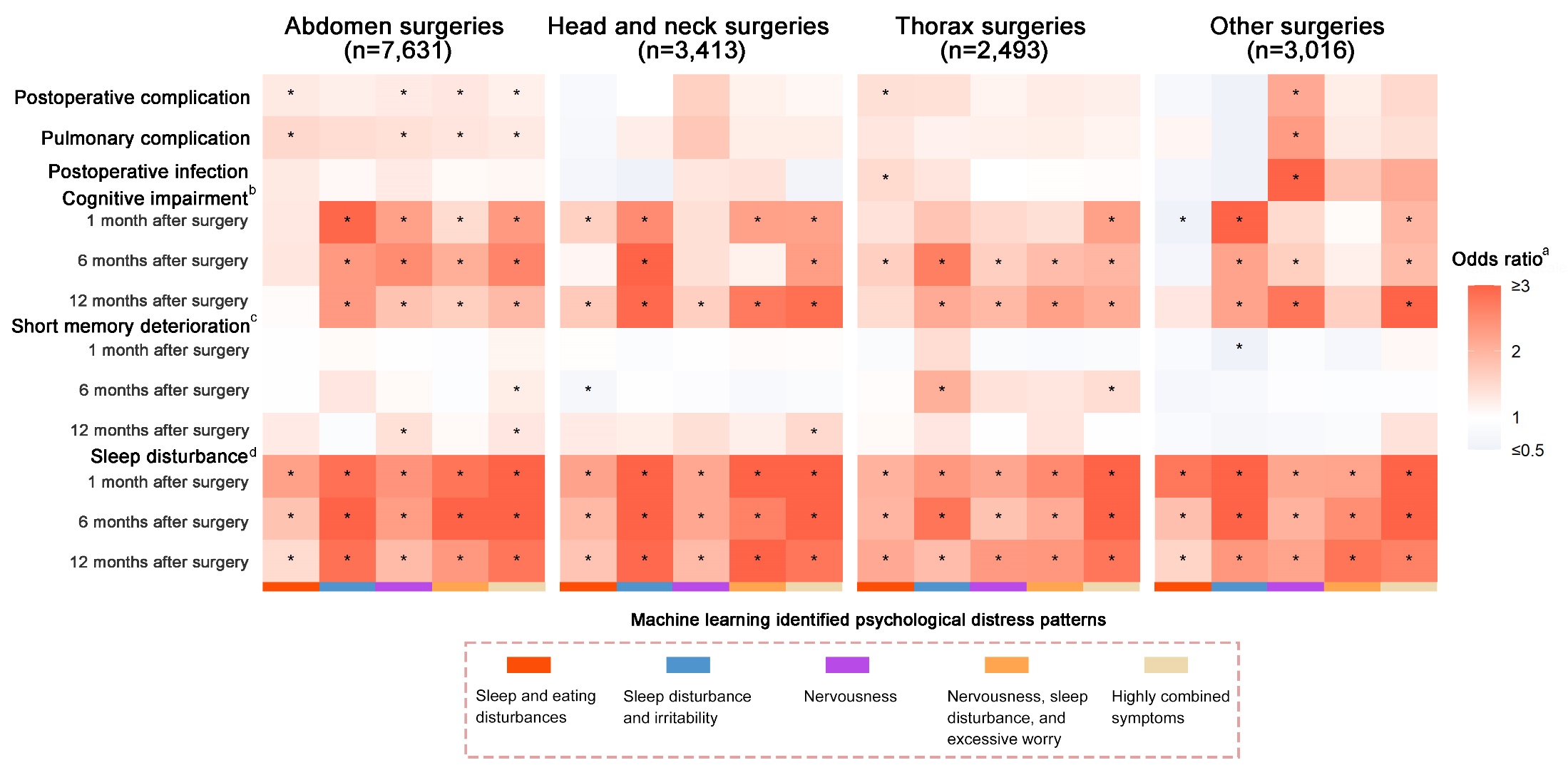
**

## Figure S2 Associations between preoperative psychopathology and adverse surgery-related binary outcomes, by surgery sites

^a^The odds ratios were derived from logistic regression models, adjusted for age, sex, body mass index, Charlson comorbidity index, smoking and drinking status, education level, and duration of surgery. The reference group was the individuals within the normal psychological functioning group. Asterisks indicate a two-sided p-value less than 0.05.

^b^Cognitive dysfunction was defined as a total score of less than 2 on the 8-Item Informant Interview to Differentiate Aging and Dementia (AD8).

^c^Short-term memory deterioration was defined as the inability to recall all three words in the Three-word Recall Test.

^d^Sleep disturbance was defined as a total score of greater than 5 on the Pittsburgh Sleep Quality Index (PSQI).

**
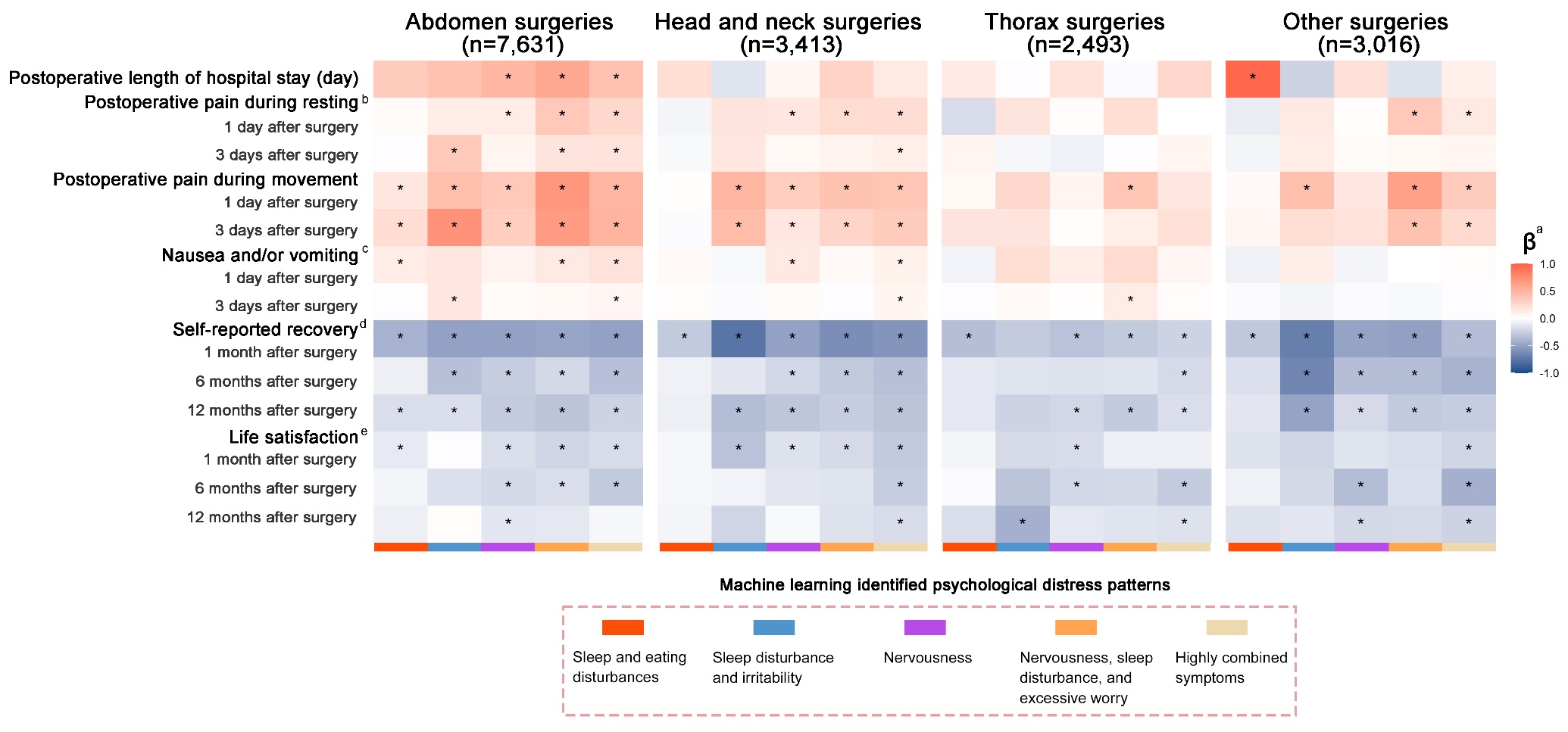
**

## Figure S3 Associations between preoperative psychopathology and adverse surgery-related continuous outcomes, by surgery sites

^a^The β were derived from linear regression models, adjusted for age, sex, body mass index, Charlson comorbidity index, smoking and drinking status, education level, and duration of surgery, all numeric value was standardized to 0-10. The reference group was the individuals within the normal psychological functioning group. Asterisks indicate a two-sided p-value less than 0.05.

^b^Pain after surgery during resting and movement were conducted at 1 day and 3 days postoperatively using an 11-point Numeric Rating Scale, with 0 indicated the absence of pain, and 10 represented the worst imaginable pain.

^c^Nausea and vomiting symptoms after surgery were conducted at 1 day and 3 days postoperatively using an 11-point Numeric Rating Scale, with 0 indicated the absence symptoms of nausea/vomiting, and 10 represented the worst imaginable symptoms of nausea/vomiting.

^d^Self-reported postoperative recovery was employed using an 11-point NRS, where a score of 0 represented the worst recovery, and 10 indicated perfect recovery.

^e^Life satisfaction was assessed using a 4-point NRS, with a score of 0 indicating total dissatisfaction and 4 indicating total satisfaction

## Supplementary table 1 Short-, intermediate- and long-term outcomes checklist

| Category | Assessment instrument | Detail of assessment |
| --- | --- | --- |
| *Short term outcomes* | | |
| Complication   - Any - Pulmonary complication - Postoperative infection | Medical records | ‘Any’ was defined as followed symptoms1-23 which ever occurred:   1. Death 2. Non-fatal cardiac arrest 3. Myocardial infarction 4. Congestive heart failure 5. Emerging arrhythmia 6. Angina pectoris 7. Respiratory tract infections 8. Respiratory failure 9. Pleural effusion 10. Atelectasis of lung 11. Pneumothorax 12. Bronchospasm 13. Aspiration pneumonia 14. Pneumonia 15. Surgical site infection 16. Urinary tract infections 17. Blood borne infections 18. Acute Kidney Injury 19. Acute respiratory distress syndrome 20. Stroke 21. Pulmonary embolism 22. Anastomotic leak 23. Cardiac arrest   ‘Pulmonary complication’ was defined as symptoms 7-13 which ever occurred.  ‘Postoperative infection’ was defined as symptoms 14-17 which ever occurred. |
| Length of hospital stay | Medical records | Time interval (days) between surgery and discharge from the hospital |
| Pain during resting | Numeric Rating Scale | Conducted at 1 day and 3 days postoperatively using an 11-point Numeric Rating Scale, with 0 indicated the absence of pain, and 10 represented the worst imaginable pain |
| Pain during movement | Numeric Rating Scale | Conducted at 1 day and 3 days postoperatively using an 11-point Numeric Rating Scale, with 0 indicated the absence of pain, and 10 represented the worst imaginable pain |
| Nausea and vomiting | Numeric Rating Scale | Conducted at 1 day and 3 days postoperatively using an 11-point Numeric Rating Scale, with 0 indicated the absence of nausea/vomiting, and 10 represented the worst imaginable nausea/vomiting |
| *Intermediate- and long-term outcomes* | | |
| Cognitive dysfunction | 8-Item Informant Interview to Differentiate Aging and Dementia, AD8 | Cognitive dysfunction was defined as a total score<2 on the AD8 |
| Short memory deterioration | Three-words recall test | Short memory deterioration defined by the inability to recall all three words in the test |
| Sleep disturbance | Pittsburgh Sleep Quality Index, PSQI | Sleep disturbance is defined as a total of score of greater than 5 on the PSQI |
| Self-reported recovery | Numeric Rating Scale | A score of 0 represented the lowest level of self-rated recovery, and 10 indicated perfect recovery. |
| Life satisfaction | Numeric Rating Scale | A score of 0 indicating total dissatisfaction and 4 indicating total satisfaction |

CD-CMS, Cohort Data Collection and Management System; AIMS, Anesthesia Information Management System

## Supplementary table 2. Characteristics of the patients, by study center

| Characteristics | West-China Hospital N=12,175 | West-China Tianfu hospital  N=3748 | The Second Hospital of Hebei Medical University  N=251 | The First People's hospital of Longquanyi District  N=379 |
| --- | --- | --- | --- | --- |
| Age at baseline (year), mean (SD) | 52.21 (7.05) | 52.44 (7.28) | 53.54 (6.59) | 51.58 (5.49) |
| Female, (%) | 7,064 (58.0) | 2,214 (59.1) | 129 (51.4) | 241 (63.6) |
| Body mass index (kg/m^2^), n (%) |  |  |  |  |
| ＜18.5 | 440 (3.6) | 112 (3.0) | 2 (0.8) | 5 (1.3) |
| 18.5-25 | 8,343 (68.5) | 2,472 (66.0) | 106 (42.2) | 214 (56.5) |
| 25-30 | 3,098 (25.4) | 1,011 (27.0) | 120 (47.8) | 136 (35.9) |
| ≥30 | 294 (2.4) | 153 (4.1) | 23 (9.2) | 24 (6.3) |
| Smoking status, n (%) |  |  |  |  |
| Never | 8,858 (72.8) | 2,827 (75.4) | 188 (74.9) | 285 (75.2) |
| Previous smoker | 996 (8.2) | 349 (9.3) | 7 (2.8) | 19 (5.0) |
| Current smoker | 2,311 (19.0) | 572 (15.3) | 56 (22.3) | 75 (19.8) |
| Unknown | 10 (0.1) | 0 (0.0) | 0 (0.0) | 0 (0.0) |
| Current alcohol consumption, n (%) |  |  |  |  |
| No | 9,782 (80.3) | 3,254 (86.8) | 198 (78.9) | 316 (83.4) |
| Yes | 2,393 (19.7) | 494 (13.2) | 53 (21.1) | 63 (16.6) |
| Marital status, n (%) |  |  |  |  |
| Unmarried | 139 (1.1) | 50 (1.3) | 4 (1.6) | 2 (0.5) |
| Married | 11,497 (94.4) | 3,608 (96.3) | 246 (98.0) | 357 (94.2) |
| Divorced/Widowed | 539 (4.4) | 90 (2.4) | 1 (0.4) | 20 (5.3) |
| Education level, n (%) |  |  |  |  |
| Elementary and lower | 207 (1.7) | 150 (4.0) | 34 (13.5) | 111 (29.3) |
| Junior school | 2,684 (22.0) | 1,206 (32.2) | 105 (41.8) | 178 (47.0) |
| Senior/Secondary school | 2,759 (22.7) | 754 (20.1) | 73 (29.1) | 44 (11.6) |
| Collage and above | 6,525 (53.6) | 1,638 (43.7) | 39 (15.5) | 46 (12.1) |
| Occupation^a^, n (%) |  |  |  |  |
| Manual laborers | 943 (7.7) | 458 (12.2) | 124 (49.4) | 82 (21.6) |
| Intellectual laborers | 5,079 (41.7) | 1,263 (33.7) | 60 (23.9) | 69 (18.2) |
| Freelancer, self-employed | 1,662 (13.7) | 520 (13.9) | 41 (16.3) | 43 (11.3) |
| Retired | 3,639 (29.9) | 1,180 (31.5) | 15 (6.0) | 45 (11.9) |
| Unemployed | 852 (7.0) | 327 (8.7) | 11 (4.4) | 140 (36.9) |
| Type of anesthesia, n (%) |  |  |  |  |
| Combined intravenous and inhalation anesthesia | 11,449 (94.0) | 3,603 (96.1) | 241 (96.0) | 333 (87.9) |
| Total intravenous anesthesia | 612 (5.0) | 128 (3.4) | 2 (0.8) | 10 (2.6) |
| Inhalation anesthesia | 94 (0.8) | 3 (0.1) | 0 (0.0) | 12 (3.2) |
| Unknown | 20 (0.2) | 14 (0.4) | 8 (3.2) | 24 (6.3) |
| Anesthesia duration, n (%) |  |  |  |  |
| ≤ 90 minutes | 2,293 (18.8) | 1034 (27.6) | 20 (8.0) | 165 (43.5) |
| 90~120 minutes | 2,399 (19.7) | 692 (18.5) | 48 (19.1) | 64 (16.9) |
| 121~180 minutes | 3,594 (29.5) | 1,121 (29.9) | 83 (33.1) | 44 (11.6) |
| >180 minutes | 3,842 (31.6) | 888 (23.7) | 93 (37.1) | 81 (21.4) |
| Unknown | 47 (0.4) | 13 (0.3) | 7 (2.8) | 25 (6.6) |
| Surgery duration, n (%) |  |  |  |  |
| ≤ 90 minutes | 6,047 (49.7) | 2,081 (55.5) | 109 (43.4) | 256 (67.5) |
| 90~120 minutes | 2,079 (17.1) | 638 (17.0) | 39 (15.5) | 32 (8.4) |
| 121~180 minutes | 2,275 (18.7) | 642 (17.1) | 44 (17.5) | 41 (10.8) |
| >180 minutes | 1,743 (14.3) | 374 (10.0) | 52 (20.7) | 25 (6.6) |
| Unknown | 31 (0.3) | 13 (0.3) | 7 (2.8) | 25 (6.6) |
| Site of surgery, n (%) |  |  |  |  |
| Abdomen | 5,566 (45.7) | 1,616 (43.1) | 146 (58.2) | 303 (79.9) |
| Head and neck | 2,631 (21.6) | 780 (20.8) | 0 (0.0) | 2 (0.5) |
| Thorax | 2,078 (17.1) | 392 (10.5) | 0 (0.0) | 23 (6.1) |
| Others | 1,900 (15.6) | 960 (25.6) | 105 (41.8) | 51 (13.5) |
| Postoperative ICU stay, n (%) |  |  |  |  |
| No | 11,938 (98.1) | 3,722 (99.3) | 230 (91.6) | 347 (91.6) |
| Yes | 232 (1.9) | 26 (0.7) | 3 (1.2) | 21 (5.5) |
| unknown | 5 (0.0) | 0 (0.0) | 18 (7.2) | 11 (2.9) |
| Postoperative length of hospital stay(day), mean (SD) | 4.04 (4.25) | 4.20 (9.02) | 6.25 (4.13) | 4.38 (2.50) |

a, Occupation was classed into 5 categories according to self-reported questionnaire: ‘Manual laborers’ incudes all workers and farmers; ‘Intellectual laborers’ includes students, civil servants, technicists, office clerks, managers, and servicemen; ‘Freelancer and self-employed’ includes freelancers and the self-employed individuals; ‘Retired’ refers solely to those who are retired; ‘Unemployed’ refers to those who are currently without a job.

b, Depression or anxiety symptoms were defined as Patient Health Questionnaire-9 (PHQ-9) total score ≥10 or Generalized Anxiety Disorder Scale-7 (GAD-7) total score ≥10.

## Supplementary table 3 Evaluation of applied clustering methods

| **Clustering Methods** | **Silhouette Score** | **Dunn’s index** |
| --- | --- | --- |
| UMAP enhanced HDBSCAN | 0.42 | 0.37 |
| K-means | 0.26 | 0.33 |
| Hierarchical clustering | 0.27 | 0.30 |

## Supplementary table 4 Characteristics of the patients, by preoperative psychiatric distress patterns

| **Postoperative characteristic** | **Machine learning-identified psychological distress patterns** | | | | | | **Cut-off based psychological symptoms** | |
| --- | --- | --- | --- | --- | --- | --- | --- | --- |
|  | **Normal psychological functioning (n=5,412)** | **Nervousness (n=2,310)** | **Nervousness, sleep disturbance, and excessive worry (n=1,336)** | **Sleep and eating disturbances (n=2,187)** | **Sleep disturbance and irritability (n=534)** | **Highly combined symptoms (n=4,407)** | **Normal**  **(n=15,920)** | **Psychological symptom**  **(n=266)** |
| **Length of hospital stay**(day), mean(sd) | 3.94 (6.39) | 4.18 (4.13) | 4.07 (3.41) | 4.35 (8.71) | 4.00 (3.33) | 4.19 (4.21) | 4.11 (5.72) | 4.64 (4.29) |
| **Any postoperative complicatio**n, n(%) |  |  |  |  |  |  |  |  |
| No | 4,879 (90.2) | 2,031 (87.9) | 1,183 (88.5) | 1,927 (88.1) | 475 (89.0) | 3,913 (88.8) | 14,175 (89.0) | 233 (87.6) |
| Yes | 521 (9.6) | 275 (11.9) | 151 (11.3) | 256 (11.7) | 57 (10.7) | 485 (11.0) | 1716 (10.8) | 25 (9.4) |
| Missing | 12 (0.2) | 4 (0.2) | 2 (0.1) | 4 (0.2) | 2 (0.4) | 9 (0.2) | 29 (0.2) | 4 (1.5) |
| **Pulmonary complication**, n(%) |  |  |  |  |  |  |  |  |
| No | 4,991 (92.2) | 2,073 (89.7) | 1,214 (90.9) | 1,962 (89.7) | 483 (90.4) | 4,004 (90.9) | 14490 (91.0) | 237 (89.1) |
| Yes | 409 (7.6) | 233 (10.1) | 120 (9.0) | 221 (10.1) | 49 (9.2) | 394 (8.9) | 1401 (8.8) | 25 (9.4) |
| Missing | 12 (0.2) | 4 (0.2) | 2 (0.1) | 4 (0.2) | 2 (0.4) | 9 (0.2) | 29 (0.2) | 4 (1.5) |
| **Postoperative infection**, n(%) |  |  |  |  |  |  |  |  |
| No | 5,115 (94.5) | 2,156 (93.3) | 1,260 (94.3) | 2,038 (93.2) | 503 (94.2) | 4147 (94.1) | 14972 (94.0) | 247 (92.9) |
| Yes | 285 (5.3) | 150 (6.5) | 74 (5.5) | 145 (6.6) | 29 (5.4) | 251 (5.7) | 919 (5.8) | 15 (5.6) |
| Missing | 12 (0.2) | 4 (0.2) | 2 (0.1) | 4 (0.2) | 2 (0.4) | 9 (0.2) | 29 (0.2) | 4 (1.5) |
| **Pain during resting**, mean(sd) |  |  |  |  |  |  |  |  |
| 1 day after surgery | 0.46 (0.65) | 0.50 (0.66) | 0.59 (0.72) | 0.44 (0.62) | 0.52 (0.68) | 0.54 (0.69) | 0.49 (0.66) | 0.68 (0.80) |
| 3 days after surgery | 0.19 (0.45) | 0.22 (0.49) | 0.25 (0.52) | 0.19 (0.45) | 0.31 (0.56) | 0.27 (0.52) | 0.22 (0.48) | 0.39 (0.66) |
| **Pain during movement**, mean(sd) |  |  |  |  |  |  |  |  |
| 1 day after surgery | 1.02 (0.75) | 1.11 (0.76) | 1.23 (0.78) | 1.06 (0.74) | 1.18 (0.76) | 1.16 (0.75) | 1.10 (0.75) | 1.28 (0.80) |
| 3 days after surgery | 0.75 (0.73) | 0.86 (0.73) | 0.93 (0.77) | 0.82 (0.76) | 0.93 (0.77) | 0.90 (0.76) | 0.83 (0.75) | 1.03 (0.79) |
| **Nausea and vomiting**, mean(sd) |  |  |  |  |  |  |  |  |
| 1 day after surgery | 0.13 (0.46) | 0.18 (0.54) | 0.21 (0.60) | 0.17 (0.52) | 0.21 (0.56) | 0.21 (0.58) | 0.17 (0.53) | 0.31 (0.72) |
| 3 days after surgery | 0.05 (0.27) | 0.06 (0.29) | 0.06 (0.32) | 0.05 (0.28) | 0.08 (0.34) | 0.07 (0.34) | 0.06 (0.30) | 0.12 (0.38) |
| 1 month follow-up |  |  |  |  |  |  |  |  |
| **Cognitive dysfunction**, n(%) |  |  |  |  |  |  |  |  |
| No | 4,840 (89.4) | 2,016 (87.3) | 1,186 (88.8) | 1,964 (89.8) | 437 (81.8) | 3,702 (84.0) | 13951 (87.6) | 194 (72.9) |
| Yes | 234 (4.3) | 185 (8.0) | 98 (7.3) | 124 (5.7) | 61 (11.4) | 436 (9.9) | 1090 (6.8) | 48 (18.0) |
| Missing | 338 (6.2) | 109 (4.7) | 52 (3.9) | 99 (4.5) | 36 (6.7) | 269 (6.1) | 879 (5.5) | 24 (9.0) |
| **Short memory deterioration**, n(%) |  |  |  |  |  |  |  |  |
| No | 2,932 (54.2) | 1,346 (58.3) | 812 (60.8) | 1,219 (55.7) | 294 (55.1) | 2,365 (53.7) | 8836 (55.5) | 132 (49.6) |
| Yes | 2,095 (38.7) | 833 (36.1) | 450 (33.7) | 848 (38.8) | 192 (36.0) | 1,726 (39.2) | 6038 (37.9) | 106 (39.8) |
| Missing | 385 (7.1) | 131 (5.7) | 74 (5.5) | 120 (5.5) | 48 (9.0) | 316 (7.2) | 1046 (6.6) | 28 (10.5) |
| **Sleep disturbance**, n(%) |  |  |  |  |  |  |  |  |
| No | 3,011 (55.6) | 834 (36.1) | 429 (32.1) | 779 (35.6) | 161 (30.1) | 1,136 (25.8) | 6314 (39.7) | 36 (13.5) |
| Yes | 2,089 (38.6) | 1,377 (59.6) | 865 (64.7) | 1,313 (60.0) | 340 (63.7) | 3,021 (68.6) | 8796 (55.3) | 209 (78.6) |
| Missing | 312 (5.8) | 99 (4.3) | 42 (3.1) | 95 (4.3) | 33 (6.2) | 250 (5.7) | 810 (5.1) | 21 (7.9) |
| **Self-reported recovery**, mean(sd) | 7.63 (1.50) | 7.13 (1.53) | 7.11 (1.53) | 7.25 (1.57) | 7.04 (1.64) | 7.15 (1.63) | 7.32 (1.57) | 6.72 (1.77) |
| **Life Satisfaction**, mean(sd) | 3.11 (0.46) | 3.05 (0.50) | 3.04 (0.51) | 3.08 (0.49) | 3.06 (0.54) | 3.04 (0.54) | 3.07 (0.50) | 2.96 (0.60) |
| 6 months follow-up |  |  |  |  |  |  |  |  |
| **Cognitive dysfunction**, n(%) |  |  |  |  |  |  |  |  |
| No | 3,867 (71.5) | 1,625 (70.3) | 971 (72.7) | 1,621 (74.1) | 359 (67.2) | 2,821 (64.0) | 11100 (69.7) | 164 (61.7) |
| Yes | 318 (5.9) | 285 (12.3) | 149 (11.2) | 175 (8.0) | 89 (16.7) | 584 (13.3) | 1551 (9.7) | 49 (18.4) |
| Missing | 1,227 (22.7) | 400 (17.3) | 216 (16.2) | 391 (17.9) | 86 (16.1) | 1,002 (22.7) | 3269 (20.5) | 53 (19.9) |
| **Short memory deterioration**, n(%) |  |  |  |  |  |  |  |  |
| No | 2,288 (42.3) | 1,013 (43.9) | 630 (47.2) | 960 (43.9) | 239 (44.8) | 1,724 (39.1) | 6752 (42.4) | 102 (38.3) |
| Yes | 1,808 (33.4) | 856 (37.1) | 467 (35.0) | 807 (36.9) | 204 (38.2) | 1,612 (36.6) | 5650 (35.5) | 104 (39.1) |
| Missing | 1,316 (24.3) | 441 (19.1) | 239 (17.9) | 420 (19.2) | 91 (17.0) | 1,071 (24.3) | 3518 (22.1) | 60 (22.6) |
| **Sleep disturbance**, n(%) |  |  |  |  |  |  |  |  |
| No | 2,839 (52.5) | 927 (40.1) | 469 (35.1) | 921 (42.1) | 178 (33.3) | 1,202 (27.3) | 6483 (40.7) | 53 (19.9) |
| Yes | 1,341 (24.8) | 983 (42.6) | 647 (48.4) | 875 (40.0) | 271 (50.7) | 2,202 (50.0) | 6158 (38.7) | 161 (60.5) |
| Missing | 1,232 (22.8) | 400 (17.3) | 220 (16.5) | 391 (17.9) | 85 (15.9) | 1,003 (22.8) | 3279 (20.6) | 52 (19.5) |
| **Self-reported recovery**, mean(sd) | 8.74 (1.15) | 8.47 (1.20) | 8.48 (1.25) | 8.61 (1.18) | 8.41 (1.44) | 8.39 (1.34) | 8.56 (1.23) | 8.11 (1.63) |
| **Life Satisfaction**, mean(sd) | 3.23 (0.57) | 3.15 (0.63) | 3.17 (0.63) | 3.21 (0.60) | 3.17 (0.62) | 3.11 (0.64) | 3.18 (0.61) | 2.88 (0.70) |
| 12 months follow-up |  |  |  |  |  |  |  |  |
| **Cognitive dysfunction**, n (%) |  |  |  |  |  |  |  |  |
| No | 2,824 (52.2) | 1,255 (54.3) | 750 (56.1) | 1,227 (56.1) | 311 (58.2) | 2,204 (50.0) | 8451 (53.1) | 120 (45.1) |
| Yes | 294 (5.4) | 272 (11.8) | 166 (12.4) | 180 (8.2) | 81 (15.2) | 566 (12.8) | 1506 (9.5) | 53 (19.9) |
| Missing | 2,294 (42.4) | 783 (33.9) | 420 (31.4) | 780 (35.7) | 142 (26.6) | 1637 (37.1) | 5963 (37.5) | 93 (35.0) |
| **Short memory deterioration**, n (%) |  |  |  |  |  |  |  |  |
| No | 1,364 (25.2) | 599 (25.9) | 376 (28.1) | 526 (24.1) | 165 (30.9) | 1,042 (23.6) | 4012 (25.2) | 60 (22.6) |
| Yes | 1,704 (31.5) | 907 (39.3) | 530 (39.7) | 850 (38.9) | 221 (41.4) | 1,670 (37.9) | 5772 (36.3) | 110 (41.4) |
| Missing | 2,344 (43.3) | 804 (34.8) | 430 (32.2) | 811 (37.1) | 148 (27.7) | 1,695 (38.5) | 6136 (38.5) | 96 (36.1) |
| **Sleep disturbance**, n(%) |  |  |  |  |  |  |  |  |
| No | 1,985 (36.7) | 686 (29.7) | 347 (26.0) | 696 (31.8) | 155 (29.0) | 1,039 (23.6) | 4860 (30.5) | 48 (18.0) |
| Yes | 1,135 (21.0) | 846 (36.6) | 568 (42.5) | 711 (32.5) | 236 (44.2) | 1,717 (39.0) | 5091 (32.0) | 122 (45.9) |
| Missing | 2,292 (42.4) | 778 (33.7) | 421 (31.5) | 780 (35.7) | 143 (26.8) | 1,651 (37.5) | 5969 (37.5) | 96 (36.1) |
| **Self-reported recovery**, mean(sd) | 8.67 (0.98) | 8.40 (1.10) | 8.36 (1.03) | 8.51 (1.03) | 8.39 (1.28) | 8.41 (1.09) | 8.50 (1.06) | 8.21 (1.22) |
| **Life Satisfaction**, mean(sd) | 3.14 (0.45) | 3.09 (0.44) | 3.08 (0.42) | 3.10 (0.46) | 3.09 (0.49) | 3.09 (0.47) | 3.11 (0.46) | 3.01 (0.46) |

Pain after surgery during resting and movement were conducted at 1 day and 3 days postoperatively using an 11-point Numeric Rating Scale, with 0 indicated the absence of pain, and 10 represented the worst imaginable pain.

Nausea and vomiting symptoms after surgery were conducted at 1 day and 3 days postoperatively using an 11-point Numeric Rating Scale, with 0 indicated the absence symptoms of nausea/vomiting, and 10 represented the worst imaginable symptoms of nausea/vomiting.

Cognitive dysfunction was defined as a total score of less than 2 on the 8-Item Informant Interview to Differentiate Aging and Dementia (AD8).

Short-term memory deterioration was defined as the inability to recall all three words in the Three-word Recall Test.

Sleep disturbance was defined as a total score of greater than 5 on the Pittsburgh Sleep Quality Index (PSQI).

Self-reported recovery was employed using an 11-point NRS, where a score of 0 represented the worst recovery, and 10 indicated perfect recovery.

Life satisfaction was assessed using a 4-point NRS, with a score of 0 indicating total dissatisfaction and 4 indicating total satisfaction

## Supplementary table 5 Sex specific characteristics of preoperative psychiatric distress patterns

| **Preoperative psychopathology** | **Female (N=9,648)** | **Male (N=6,905)** | | **Overall (N=16,553)** | |
| --- | --- | --- | --- | --- | --- |
| ***Machine learning-identified psychological distress patterns, n (%)*** | | | | | |
| Normal psychological functioning | 2,604 (27.7) | | 2,808 (41.4) | | 5,412 (33.4) |
| Nervousness | 1,419 (15.1) | | 891 (13.2) | | 2,310 (14.3) |
| Nervousness, sleep disturbance, and excessive worry | 950 (10.1) | | 386 (5.7) | | 1,336 (8.3) |
| Sleep and eating disturbances | 1,289 (13.7) | | 898 (13.3) | | 2,187 (13.5) |
| Sleep disturbance and irritability | 325 (3.5) | | 209 (3.1) | | 534 (3.3) |
| Highly combined symptoms | 2,824 (30.0) | | 1,583 (23.4) | | 4,407 (27.2) |
| ***Cut-off based psychological symptoms, n (%)*** | | | | | |
| Normal | 9,457 (98.0) | | 6,816 (98.7) | | 16,273 (98.3) |
| Psychological symptom | 191 (2.0) | | 89 (1.3) | | 280 (1.7) |

Cut-off based psychological symptoms were defined as Patient Health Questionnaire-9 (PHQ-9) total score ≥10 or Generalized Anxiety Disorder Scale-7 (GAD-7) total score ≥10.

There were 367 patients that recognized as the outliers in machine learning-identified psychological distress patterns (i.e., did not have to belong to any identified pattern)
